# The *Candida albicans* HIR histone chaperone regulates the yeast-to-hyphae transition by controlling the sensitivity to morphogenesis signals

**DOI:** 10.1038/s41598-017-08239-9

**Published:** 2017-08-16

**Authors:** Sabrina Jenull, Michael Tscherner, Megha Gulati, Clarissa J. Nobile, Neeraj Chauhan, Karl Kuchler

**Affiliations:** 1 Medical University of Vienna, Max F. Perutz Laboratories, Department of Medical Biochemistry, Campus Vienna Biocenter, Dr.-Bohr-Gasse 9/2, A-1030 Vienna, Austria; 2 Department of Molecular and Cell Biology, School of Natural Sciences, University of California-Merced, Merced, CA USA; 3 Public Health Research Institute, Department of Microbiology, Biochemistry and Molecular Genetics, New Jersey Medical School – Rutgers, The State University of New Jersey, Newark, New Jersey USA; 4 Department of Systems Biology, Harvard Medical School, Boston, Massachusetts 02115 USA

## Abstract

Morphological plasticity such as the yeast-to-hyphae transition is a key virulence factor of the human fungal pathogen *Candida albicans*. Hyphal formation is controlled by a multilayer regulatory network composed of environmental sensing, signaling, transcriptional modulators as well as chromatin modifications. Here, we demonstrate a novel role for the replication-independent HIR histone chaperone complex in fungal morphogenesis. HIR operates as a crucial modulator of hyphal development, since genetic ablation of the HIR complex subunit Hir1 decreases sensitivity to morphogenetic stimuli. Strikingly, *HIR1*-deficient cells display altered transcriptional amplitudes upon hyphal initiation, suggesting that Hir1 affects transcription by establishing transcriptional thresholds required for driving morphogenetic cell-fate decisions. Furthermore, ectopic expression of the transcription factor Ume6, which facilitates hyphal maintenance, rescues filamentation defects of *hir1*Δ/Δ cells, suggesting that Hir1 impacts the early phase of hyphal initiation. Hence, chromatin chaperone-mediated fine-tuning of transcription is crucial for driving morphogenetic conversions in the fungal pathogen *C*. *albicans*.

## Introduction

Chromatin plays fundamental roles in gene regulation during most cellular differentiation processes. Of note, chromatin architecture and functions have been conserved from human embryonic development to morphogenetic cell fate decisions in unicellular eukaryotes including the fungal pathogen *Candida albicans* ^1–3^. *C*. *albicans*, a normal commensal colonizer of most healthy individuals, is present on skin, gut and mucosal surfaces, and thus well-adapted to host niches with distinct immune surveillance^4–6^. However, immunosuppression can trigger *C*. *albicans* to switch into an invasive pathogenic mode, causing more than 400,000 life-threating invasive infections worldwide per year. Remarkably, together with other major pathogens, fungal infections claim about 1.5 million lives each year^7^.

The ability to undergo morphogenesis arguably constitutes one of the major virulence traits of *C*. *albicans*. The genomic plasticity is manifested by a transcriptional plasticity that enables a switch between a unicellular yeast-like morphology and the pseudohyphal/hyphal growth phases, which display characteristic septated filaments^8–10^. Hyphal formation is triggered by various environmental host signals. For instance, elevated temperatures, serum, nutrient availability, carbon dioxide, certain amino acids or N-acetyl glucosamine (GlcNAc) activate a complex signaling network, including the mitogen-activated protein kinase (MAPK) cascades and the cyclic adenosine monophosphate (cAMP)/protein kinase A (PKA) signaling pathway, resulting in the activation of a panel of dedicated transcription factors such as Efg1^9, 11–15^. Of note, besides the induction of transcriptional activators, hyphal triggers occlude transcriptional repressors of hyphal-specific genes (HSGs) such as Nrg1 from target promoters^16^. To sustain hyphal development, additional transcription factors such as Ume6 and Brg1 are recruited to HSGs, whereas prolonged binding of Nrg1 is inhibited. Thus, two distinct transcriptional phases modulate filamentation, involving both decoration and exclusion of transcriptional modulators to initiate and maintain hyphal growth^3, 17^.

Recent studies suggest a pivotal role for chromatin modification and organization in controlling fungal morphogenesis^18–20^. Moreover, Efg1 cooperates with the NuA4 histone acetyltransferase (HAT) complex and the ATP dependent chromatin remodeling complex SWI/SNF during hyphal initiation^21^. In addition, the Set3 histone deacetylase (HDAC) complex controls the threshold sensitivity of the cAMP/PKA pathway to hyphal stimuli^22^, while the Hda1 HDAC functions in prolonged hyphal growth^17^. Hence, the dynamic interplay of transcription factors and chromatin modification status integrates diverse triggers from upstream signaling cascades to set levels of coordinated transcriptional responses required for morphogenetic cell fate decisions.

The functional status of chromatin as well as its architecture can be altered by chromatin modifications including chromatin remodeling through the concomitant assembly and disassembly of nucleosomes. This process is guarded and facilitated by conserved histone chaperones acting in replication-dependent and –independent pathways^23^. The central hub for histone H3 and H4 turnover in the cytoplasm is the histone chaperone Asf1^24^. Newly synthesized histone H3/H4 dimers are acetylated by the HAT Hat1 at H4K5 and K12 and delivered to Asf1^25^. The dimer is further acetylated by the Rtt109 HAT at H3K56 in *Saccharomyces cerevisiae* ^26^. Asf1 shuttles modified H3/H4 dimers into the nucleus to a panel of other histone chaperones for chromatin assembly coupled to DNA replication, DNA damage repair, heterochromatin maintenance or transcription^24, 27^. For instance, the CAF-1 histone chaperone complex incorporates histones H3 and H4 into chromatin coupled to DNA-replication^28^. Notably, CAF-1 is involved in epigenetic white-opaque switching and in the oxidative stress response in *C*. *albicans* ^29, 30^. The HIR histone chaperone complex consists of four subunits in *S*. *cerevisiae* and assembles nucleosomes in a replication-independent manner^31–33^. The complex was originally discovered in *S*. *cerevisiae* as transcriptional repressor for 6 out of 8 histone genes^34^. Of note, functions of HIR related to non-histone gene regulation have been proposed, including cryptic promoter repression and suppression of Ty elements^32, 35^. Interestingly, loss of the HIR complex subunit Hir1 decreases nucleosome occupancy at various gene promoters^36^. Hence, Hir1 might affect the fine-tuning of transcriptional responses by regulating local chromatin architecture in target genes, thereby altering affinities of dedicated transcriptional regulators to cognate *cis*-acting sites, as well as the decoration by and displacement of transcriptional co-factors and modulators.

Initiation of *C*. *albicans* morphogenesis is accompanied by massive transcriptional changes affecting up to 15% of the genome^37^. Hence, we hypothesized that defects in replication-independent chromatin assembly may deregulate HSG expression and thus, influence the *C*. *albicans* yeast-to-hyphae transition. Indeed, here we show that chromatin assembly during filamentation is facilitated by *HIR1*, but not by the replication-dependent CAF-1 machinery. We find that HSG transcriptional amplitudes dramatically change upon genetic removal of *HIR1*. Furthermore, we provide compelling evidence that the HIR complex acts downstream of cAMP/PKA signaling, possibly in concert with other transcriptional activators such as Efg1, to regulate the initial onset of hyphal growth. Surprisingly, transcriptomics of *HIR1-*deficient cells revealed that the gene expression profile in cells lacking *HIR1* was qualitatively very similar to the wild type (WT) following hyphal induction. However, the maximal transcriptional amplitudes are remarkably altered in the *HIR1* mutant compared to the WT, which results in strongly decreased sensitivity of cells to filamentation signals. Strikingly, ectopic expression of Ume6 bypasses the requirement of Hir1-mediated transcriptional initiation for hyphal differentiation, which further substantiates our conclusion that Hir1 plays an important role during early steps of hyphal initiation. Our data are of general relevance, since they suggest a novel mechanism whereby chromatin chaperones like the HIR complex can impact fine-tuning of transcription to establish minimal threshold transcriptional levels required for triggering developmental changes or morphogenetic cell fate decisions independently of DNA replication or cell cycle control.

## Results

### The HIR complex facilitates hyphal initiation

**Figure 1:**
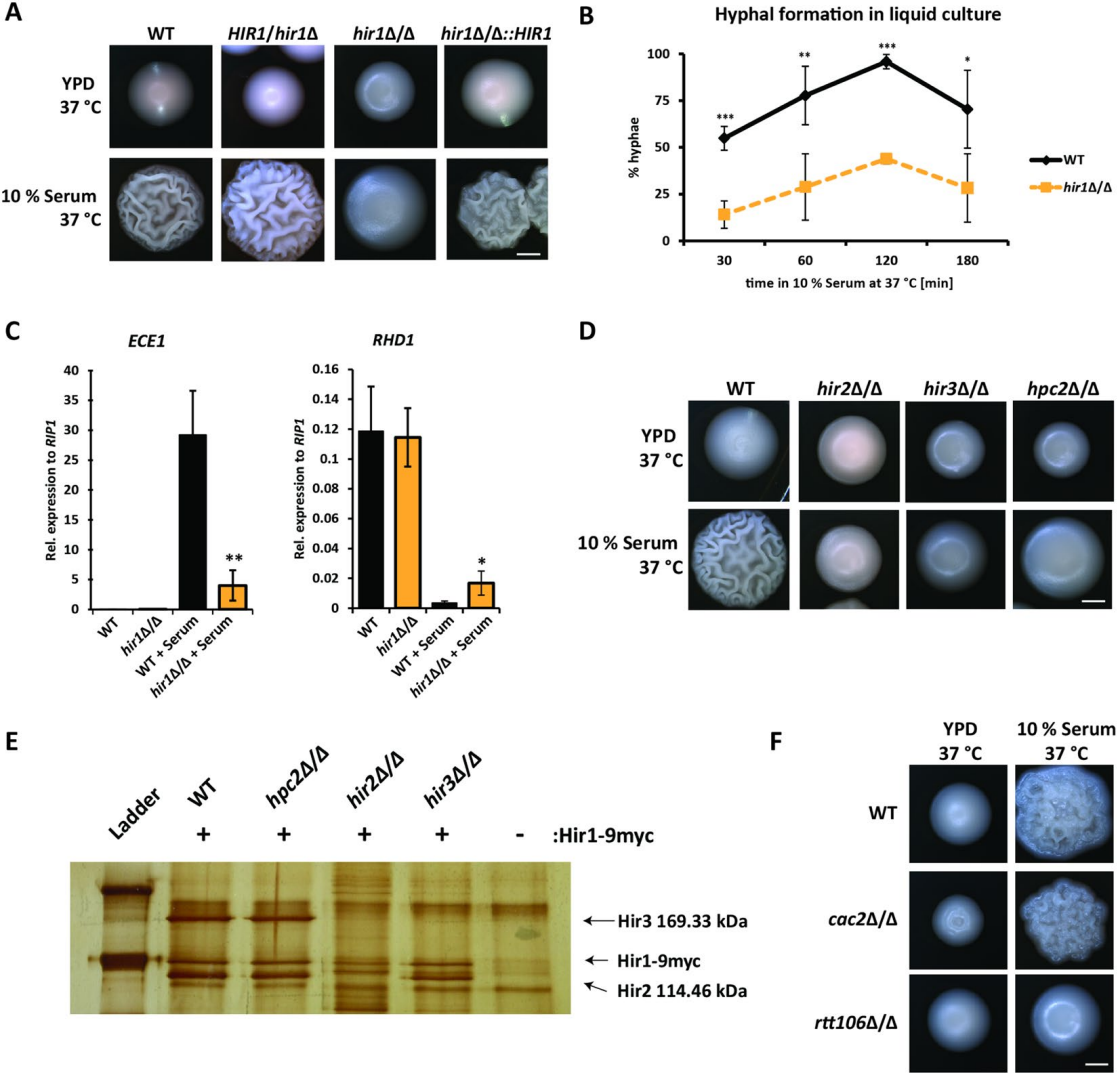
Regulation of hyphal formation by the HIR complex. (**A**) *hir1*Δ/Δ cells are defective in serum-induced hyphal formation on solid medium. Colony morphology was inspected after 3 days. Scale bar corresponds to 1 mm. (**B**) Loss of *HIR1* decreases hyphal formation in liquid medium. Hyphal formation of WT and *hir1*Δ/Δ cells was evaluated in YPD supplemented with 10% FCS at 37 °C after the indicated time points. (**C**) Hir1 controls hyphal-specific gene (HSG) expression. *ECE1* and *RHD1* gene expression was measured after 30 min of hyphal induction via RT-qPCR. Transcript levels were normalized to the expression of the reference gene *RIP1* ^22^. (**D**) HIR complex mutants display decreased hyphal formation. Colony morphology of *hpc2*Δ/Δ, *hir2*Δ/Δ and *hir3*Δ/Δ was examined as in (**A**). Scale bar corresponds to 1 mm. (**E**) Hir1 associates with Hir2 and Hir3 in *C*. *albicans*. Hir1 was epitope-tagged with 9myc in either WT background or in strains lacking one HIR complex component and subjected to native immunoprecipitation. Precipitated proteins were separated through a 10% SDS-PAGE gel and visualized by silver staining. Detected protein bands were cut out and identified via mass spectrometry analysis. The indicated molecular weight for Hir3 and Hir2 are derived from mass spectrometry analysis. (**F**) Hyphal formation is Cac2-independent. Colony morphology of WT cells and cells lacking *CAC2* and *RTT106* was analyzed as above (**A**). Scale bar corresponds to 1 mm. (**B**,**C**) Data are presented as mean + SD of three independent experiments. For significance testing, *hir1*Δ/Δ cells were compared to WT cells. n.s. not significant, *P < 0.05, **P < 0.01, ***P < 0.001 with Student’s t-test.

Several previous studies suggest that chromatin alterations contribute to yeast-to-hyphae transitions^16, 17, 19, 21, 37, 38^. Moreover, recent work also uncovered a role of the HIR histone chaperone member Hir1 in white-opaque switching^29^ and azole tolerance^30^ in *C*. *albicans*. Hence, we reasoned that a functional connection may exist between hyphal formation and chromatin assembly mediated by the HIR histone chaperone complex. Thus, we genetically removed *HIR1* ^30^ and subjected the *hir1*Δ/Δ mutant to filament-inducing conditions. Strikingly, loss of Hir1 strongly decreased hyphal formation on solid YPD containing 10% serum at 37 °C as indicated by smooth colony morphology (Fig. 1A). Reintegration of *HIR1* into its original genomic locus fully restored the WT phenotype. In addition, *hir1*Δ/Δ cells were defective for hyphal formation in response to other stimuli such as GlcNAc or Spider medium (Figure S1A). To investigate the filamentation defects of mutant cells in greater detail, we examined the hyphal initiation rates of *HIR1*-deficient cells in liquid YPD medium with 10% serum at 37 °C. The *hir1*Δ/Δ mutant showed severe hyphal formation defects at all time points, where only 30% of the *hir1*Δ/Δ cells initiated hyphal induction (Figs 1B and S1B).

Hyphal initiation is triggered by the so-called hyphal-specific transcriptional program^37^. Therefore, we examined the dynamics of HSG expression in *hir1*Δ/Δ cells using qRT-PCR. Induction of HSGs encoding the cell wall proteins Ece1 and Hwp1 or the G1-cyclin Hgc1^9^ was remarkably reduced in *hir1*Δ/Δ cells after 30 min growth in YPD plus 10% serum at 37 °C (Figs 1C and S1D). This result suggested that Hir1 is required for the activation of hyphal-specific transcriptional programs. Since hyphal induction not only drives gene activation^39^, we also tested expression of the β-mannosyltransferase Rhd1 which is normally repressed upon the hyphal switch^40^. Indeed, *RHD1* mRNA abundance was decreased during hyphal initiation in WT cells. A qualitatively similar response was observed in *hir1*Δ/Δ cells, although the amplitude of *RHD1* transcriptional repression was decreased (Fig. 1C). In addition, Ywp1, a yeast phase cell wall protein^41^, was not downregulated in *hir1*Δ/Δ cells in response to hyphal induction. Interestingly, basal *YWP1* expression was already substantially increased in *hir1*Δ/Δ cells during yeast phase growth (Figure S1D). These data strongly suggest that the transcriptional program during hyphal initiation cannot be fully activated in the absence of *HIR1*, thus impairing the yeast-to-hyphae transition. Since Hir1 might act globally during replication-independent chromatin assembly, we tested whether transcriptional amplitudes in response to other specific environmental cues are affected upon the loss of *HIR1*. Therefore, WT and *hir1*Δ/Δ cells were exposed to oxidative stress with H_2_O_2_ and the mRNA expression rate of the catalase *CAT1* ^42^ was quantified using RT-qPCR. The amplitude and kinetics of *CAT1* induction were almost identical in the WT and the *HIR1* mutant (Figure S2), showing that Hir1 function is dispensable for at least one stress response mechanism that requires rapid transcriptional reprogramming.

The HIR histone chaperone complex consists of the four subunits Hir1, Hir2, Hir3 and Hpc2^32^ encoded in the *C*. *albicans* genome^29^. The removal of complex subunits such as Hir2, Hir3 and Hpc2 phenocopied the *hir1*Δ/Δ deletion (Figs 1D and S1C). This demonstrates that the HIR complex is conserved between *S*. *cerevisiae* and *C*. *albicans*. Additionally, mass spectrometry analysis of the immunopurified HIR complex from *C*. *albicans* showed that Hir3 and Hir2 co-purify with functionally myc-tagged Hir1 (Fig. 1E). Of note, we did not detect Hpc2 in our Co-IP experimental set-up. This might be due to a low silver staining efficiency of Hpc2 as reported earlier^33^. Since the loss of each individual complex subunit had the same effect on hyphal formation, we also tested how complex formation is affected by the loss of one member. Therefore, Hir1 was myc-tagged in *hir2*Δ/Δ, *hir3*Δ/Δ and *hpc2*Δ/Δ strains and subjected to co-immunoprecipitation. Upon loss of *HPC2*, Hir2 and Hir3 remained associated with Hir1-myc, while deletion of *HIR2* abolishes binding of Hir3 to Hir1-myc. Furthermore, Hir1-myc and Hir2 interaction was still maintained in the absence of *HIR3* (Fig. 1E). These data suggest that specific HIR complex subunits may execute distinct functions, ranging from assembly (Hir2) to the recruitment to target genes (Hpc2), as previously speculated in *S*. *cerevisiae* ^35^.

Since chromatin assembly uses two distinct pathways^43^, we analyzed the specificity of the HIR complex for regulating the yeast-to-hyphae transition. Cells lacking the CAF-1 subunit Cac2 implicated in the replication-coupled chromatin assembly^44^, showed no defects in hyphal formation (Fig. 1F). By contrast, deletion of *RTT106*, a histone chaperone involved in both pathways^45^, phenocopied Hir1 ablation on solid medium, albeit filamentation defects were less pronounced than in *hir1*Δ/Δ cells, since hyphal formation was not disrupted in liquid medium (data not shown). Hence, hyphal induction rather requires mainly HIR-mediated replication-independent chromatin assembly.

### Loss of Hir1 mimics lack of Efg1

**Figure 2:**
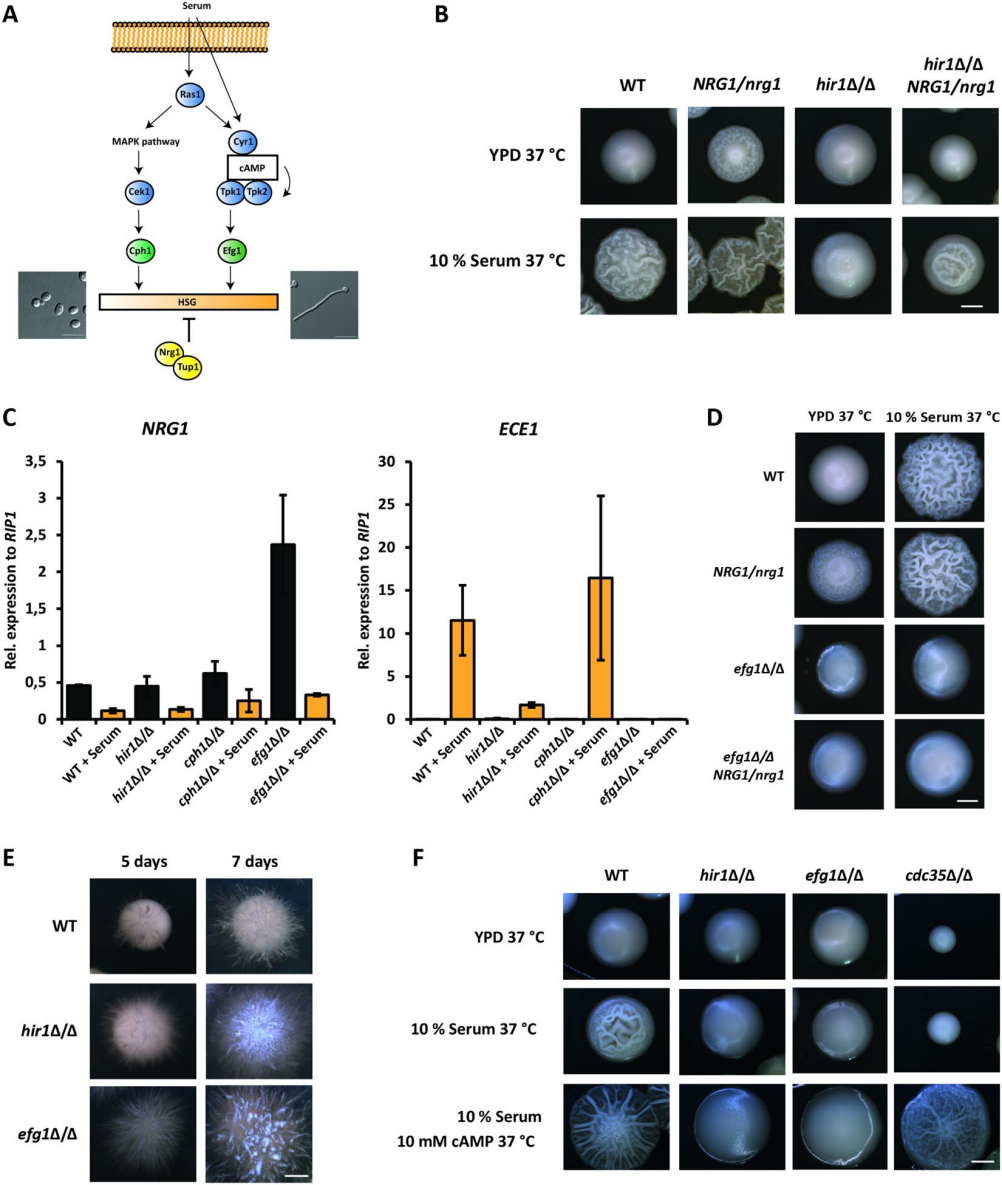
Loss of *HIR1* phenocopies deletion of *EFG1*. (**A**) Simplified scheme of the major signaling cascades regulating the hyphal transcriptional program. The MAPK cascade and the cAMP/PKA pathway activate transcription factors such as Efg1 and Cph1 to initiate HSG expression. Additionally, transcriptional repressors such as Nrg1 and Tup1 control HSG expression and are themselves regulated by upstream signaling cascades such as cAMP/PKA. (**B**) Reduction in *NRG1* gene dosage alleviates hyphal formation defects of *hir1*Δ/Δ cells on solid medium. Colony morphology was inspected after 3 days at 37 °C. Scale bar corresponds to 1 mm. (**C**) *NRG1* downregulation is independent of *EFG1*, *CPH1* and *HIR1*. Gene expression of *NRG1* and *ECE1* was measured after 30 min of hyphal induction in YPD with 10% FCS by RT-qPCR. Transcript levels were normalized to the expression of the reference gene *RIP1*. Data are presented as mean + SD of three independent experiments. (**D**) Reduction of *NRG1* gene dosage does not affect *efg1*∆/∆ hyphal formation. Colony morphology was assessed after 3 days at 37 °C. Scale bar corresponds to 1 mm. (**E**) Loss of *HIR1* partially phenocopies *efg1*Δ/Δ under embedded growth conditions. Colony pictures were taken after 5 and 7 days at 25 °C. Scale bar corresponds to 1 mm. (**F**) *HIR1* mutant cells are irresponsive to exogenous cAMP supplementation. Colony morphology was assessed after 3 days at 37 °C. Scale bar corresponds to 1 mm.

HSG induction depends on a complex signaling network. Two major signaling pathways, the cAMP/PKA and a MAPK pathway converge at the activation of downstream transcription factors such as Efg1 and Cph1^9, 46^, and relief from Tup1 and Nrg1 repression^16, 47^ as shown in Fig. 2A. In *S*. *cerevisiae*, the Hir1-containing HIR complex acts as transcriptional repressor^34, 36, 48^. Hence, we reasoned that transcriptional repressors of HSGs may become deregulated and thus overexpressed in the absence of *HIR1*. A well-characterized transcription factor for hyphal formation is Nrg1, which represses filamentation. Consequently, its removal leads to constitutive filamentation^9, 49^. First, we tested whether deletion of *HIR1* could alleviate the hyperfilamentation phenotype of *nrg1*Δ/Δ cells. Epistasis analysis of the *hir1*Δ/Δ *nrg1*Δ/Δ double mutant revealed that deletion of *HIR1* in the *nrg1*Δ/Δ background failed to ameliorate the hyperfilamentation phenotype of *nrg1*Δ/Δ cells (Figure S3A). Of note, transcriptional downregulation of *NRG1* was not impaired in *hir1*Δ/Δ cells upon hyphal triggers, whereas the activation of HSGs like *ECE1* was strongly decreased (Figure S3C). Monitoring protein levels of functionally myc-tagged Nrg1 mirrored these data. Nrg1 was degraded independently of *HIR1*, as soon as cells activated the hyphal program (Figure S3D). These results suggest that *hir1*Δ/Δ cells are still able to sense hyphal-inducing conditions as *NRG1* mRNA downregulation and Nrg1 degradation was fully functional in the mutant.

As stated before, loss of *NRG1* leads to a massive hyperfilamenting phenotype due to de-repression of HSGs. To uncover more subtle effects of possible interactions between Hir1 and Nrg1, we tested the effect of the deletion of one *NRG1* allele on Hir1-mediated filamentation. In liquid medium, *NRG1* heterozygosity in the *hir1*Δ/Δ background had no effect on hyphal formation after 60 minutes of serum-induced filamentation (Figure S3B). On solid YPD medium at 37 °C, unlike the smooth colony appearance of WT cells, *NRG1*/*nrg1* cells formed wrinkled colonies, indicating filamentous growth. This phenotype was reverted upon genetic removal of *HIR1* in the *NRG1*/*nrg1* strain (Fig. 2B). In addition, the *NRG1*/*nrg1 hir1*Δ/Δ mutant showed reduced hyphal formation in response to serum when compared to *NRG1*/*nrg1* cells (Fig. 2B). Thus, a subtle de-repression of HSGs by decreased *NRG1* gene dosage is not sufficient to trigger full filamentous growth of *HIR1*-deficient cells.

As mentioned above, major positive regulators of hyphal formation are Cph1 and Efg1, as their deletion results in hyphal formation defects^47, 50^. We speculated that Hir1 could act in concert with Cph1 or Efg1 to adjust hyphal initiation. Therefore, we directly compared the behavior of cells lacking *HIR1*, *EFG1* or *CPH1* during growth in serum-containing YPD at elevated temperatures. Like *hir1*Δ/Δ cells, *efg1Δ/Δ* and *cph1Δ/Δ* cells downregulated *NRG1* levels upon hyphal induction (Fig. 2C). However, only *hir1*Δ/Δ and *efg1*Δ/Δ knock-out cells lacked full activation of the HSG *ECE1*. Moreover, loss of *CPH1* had no influence on hyphal formation on serum-containing YPD (data not shown) or *ECE1* transcript abundance (Fig. 2C). Genetic removal of *EFG1* altered *ECE1* expression and hyphal formation more severely than a *HIR1* deletion (Fig. 2C). Of note, *efg1*Δ/Δ cells are locked in the yeast phase during serum-induced filamentation^15, 51^. Furthermore, *NRG1* heterozygosity failed to enhance hyphal growth in *efg1*Δ/Δ cells (Fig. 2D). Interestingly, *hir1*Δ/Δ cells in part phenocopied *efg1*Δ/Δ cells under embedded conditions, where Efg1 can act as a repressor of filamentation. Consequently, loss of *EFG1* results in hyperfilamentation under these conditions^52, 53^. Likewise, *HIR1*-deficient cells showed enhanced hyphal formation during embedded growth (Fig. 2E). Of note, *EFG1* transcription in yeast morphology growth was not affected by a *HIR1* deletion (Figure S4).

Because cAMP/PKA signaling is essential for hyphal formation^3, 54^, we tested the integrity of this pathway in *hir1*Δ/Δ cells. If loss of *HIR1* debilitates cAMP generation, exogenous cAMP should restore hyphal formation in *hir1*Δ/Δ cells. However, exogenous cAMP in hyphal-inducing medium failed to trigger the yeast-to-hyphae transition in *HIR1*-deficient cells or in cells lacking *EFG1* (Fig. 2F). Cdc35, also known as Cyr1, is the adenylate cyclase synthesizing cAMP^55^. Unlike *hir1*Δ/Δ and *efg1*Δ/Δ cells, hyphal defects seen in a *cdc35*Δ/Δ mutant are fully restored upon the addition of exogenous cAMP (Fig. 2F). Notably, cAMP/PKA signaling is also involved in transcriptional downregulation of Nrg1^16, 56^. Thus, *NRG1* expression analysis (Figure S3C) suggests that the unresponsiveness of *HIR1-*deficient cells to cAMP signals (Fig. 2F) cannot be a consequence of defective cAMP/PKA signaling. These results suggest that Hir1 might affect hyphal formation downstream of cAMP/PKA signaling in the same pathway as Efg1.

### *HIR1*-deficiency impacts the amplitude of hyphal-specific gene transcription

**Figure 3:**
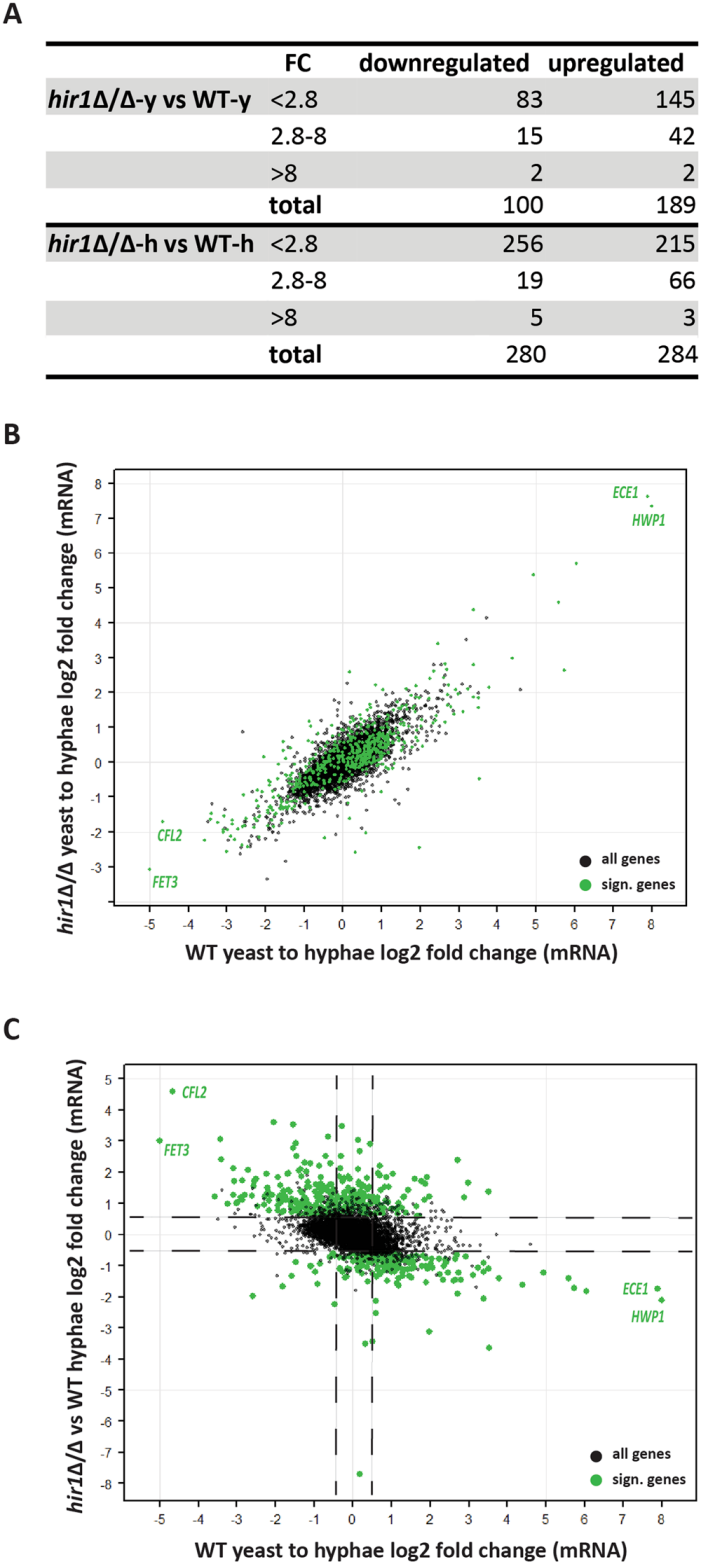
Hir1 affects the transcriptional amplitude during initiation of hyphal formation. (**A**) Absolute numbers of differentially expressed transcripts between WT and the *hir1*Δ/Δ mutant during yeast (y) or hyphal (h) growth. Differentially expressed genes were defined by FDR < 0.05. FC, fold change. (**B**) The transcriptional profile of *hir1*Δ/Δ cells showed high similarity to WT cells. log2-fold changes in RNA expression in WT cells during the yeast-to-hyphae transition (x-axis) were plotted against the log2-fold change in mRNA levels in *hir1*Δ/Δ cells during the yeast-to-hyphae transition (y-axis). (**C**) Loss of *HIR1* causes transcriptional deregulation during hyphal initiation. Each dot corresponds to one ORF. The log2-fold changes in RNA expression in WT cells during the yeast-to-hyphae transition (x-axis) were plotted against the log2-fold changes between *HIR1* mutant and WT cells in response to hyphal-inducing conditions (y-axis). (**B**,**C**) Significantly differentially expressed genes in the *hir1*Δ/Δ knock-out versus WT are depicted in green and were defined by an at least 1.5-fold change and FDR < 0.05.

We^37^ and others^40^ demonstrated that the yeast-to-hyphae transition is accompanied by massive transcriptional changes. To determine how removal of *HIR1* affects transcriptional responses, we performed RNA-seq of *hir1*Δ/Δ versus WT cells under yeast phase promoting conditions and after 30 minutes in YPD plus 10% serum at 37 °C. During yeast phase growth, some 189 genes were at least 1.5-fold upregulated, while 100 genes were downregulated to the same extent in *hir1*Δ/Δ cells when compared to WT cells. This indicates that Hir1 is acting not only as a transcriptional repressor, but is also implicated in transcriptional activation. The amplitude of the majority of differentially expressed genes covered a fold-change of 1.5 to 2.8 (Fig. 3A). Remarkably though, the number of differentially expressed genes between *hir1*Δ/Δ and WT almost doubled upon hyphal initiation when compared to the yeast phase. We found 284 genes at least 1.5-fold upregulated and 280 genes downregulated (Fig. 3A). As already observed for the yeast growth phase, the amplitude of transcriptional changes after removing *HIR1* ranged from a fold-change of 1.5 and 2.8 (Fig. 3A). These data strongly suggest that Hir1 affects fine-tuning of transcriptional amplitudes upon hyphal formation.

Detailed inspection of transcriptomes from the WT and *hir1*Δ/Δ cells during the yeast-to-hyphae transition revealed qualitatively similar transcriptional responses concerning the nature and type of genes regulated in both strains (Fig. 3B). For instance, *ECE1* and *HWP1* showed the highest induction in WT cells following hyphal growth stimulation. The same was true for *HIR1*-deficient cells (Fig. 3B, top right corner). Similarly, *FET3* and *CFL2* were the most repressed genes in the WT and *hir1*Δ/Δ mutant (Fig. 3B, bottom left corner). Remarkably, the same genes (*ECE1*, *HWP1*, *FET3*, *CFL2*) were also among the most up- as well as downregulated genes in *hir1*Δ/Δ hyphae when compared to WT hyphae (Fig. 3C). However, it was obvious that genes with increased expression in WT during the yeast-to-hyphae transition showed much lower transcript levels in the absence of *HIR1* and *vice versa*. In summary, these data demonstrate that transcriptional amplitudes of both activated and repressed HSG sets dramatically change during morphogenesis-associated transcriptional reprogramming upon loss of the Hir1 chaperone complex subunit (Fig. 3C). Hence, Hir1 can function both as transcriptional activator and repressor, thereby affecting the transcriptional fine-tuning in response to hyphal stimuli.

### The loss of *HIR1* affects different biological processes upon hyphal induction

The RNA-seq data of *hir1*Δ/Δ cells in the yeast growth phase revealed that major transcriptional changes already occurred during this growth phase (Figure S5A). Gene ontology (GO) analysis identified the gene sets affected by HIR. Differentially expressed genes in *hir1*Δ/Δ cells fall into fatty acid metabolism, cell surface remodeling and oxido-reduction processes, including genes involved in iron acquisition (*FRP1* and *CFL22*) and anti-oxidative enzymes (*SOD5* and *GPX2*). Furthermore, genes associated with DNA repeat maintenance and various transcription factors showed altered expression upon *HIR1* deletion (Figure S5A,C). Of note, less than 30% of all upregulated genes in *hir1*Δ/Δ cells (either during yeast growth or hyphal induction) were mapped to a well-characterized GO category.

Interestingly, transcriptional changes owing to the loss of *HIR1* were more severe during hyphal initiation than during the yeast phase (Fig. 3A). Further characterization of this gene set showed that upregulated transcripts encoded metabolism genes involved in glycolysis, fatty acid catabolism and the glyoxysome function (Figure S5B,D).

Downregulated genes are implicated in ER to Golgi vesicle trafficking, cell wall remodeling, filamentous growth and thus, fungal virulence (Figure S5B,D). The GO term analysis suggests that similar gene clusters are differentially regulated during *hir1*Δ/Δ yeast and hyphal growth. This was substantiated by the fact that many of the genes included in one GO group for hyphal *hir1*Δ/Δ cells were found differentially expressed in *hir1*Δ/Δ yeast growth (Figure S5A,B, gene*). However, the total number of overlapping genes with altered expression in *hir1*Δ/Δ yeast or hyphal phase is lower than suggested by the GO term analysis. Roughly one third of upregulated genes in *HIR1-*deficient hyphal cells were already elevated during yeast growth (106 out of 289). Moreover, only one fifth of all downregulated genes upon hyphal initiation in *hir1*Δ/Δ cells were also found in the yeast phase of the mutant (57 out of 280; Figure S5E,F). The majority of differentially expressed genes in *hir1*Δ/Δ cells were detected specifically upon hyphal induction. Hence, Hir1 function becomes more important for sustaining transcriptional responses to changing growth conditions, thereby affecting various biological processes, ranging from transcription control of metabolism, ER-Golgi trafficking to cell wall modulation.

**Figure 4:**
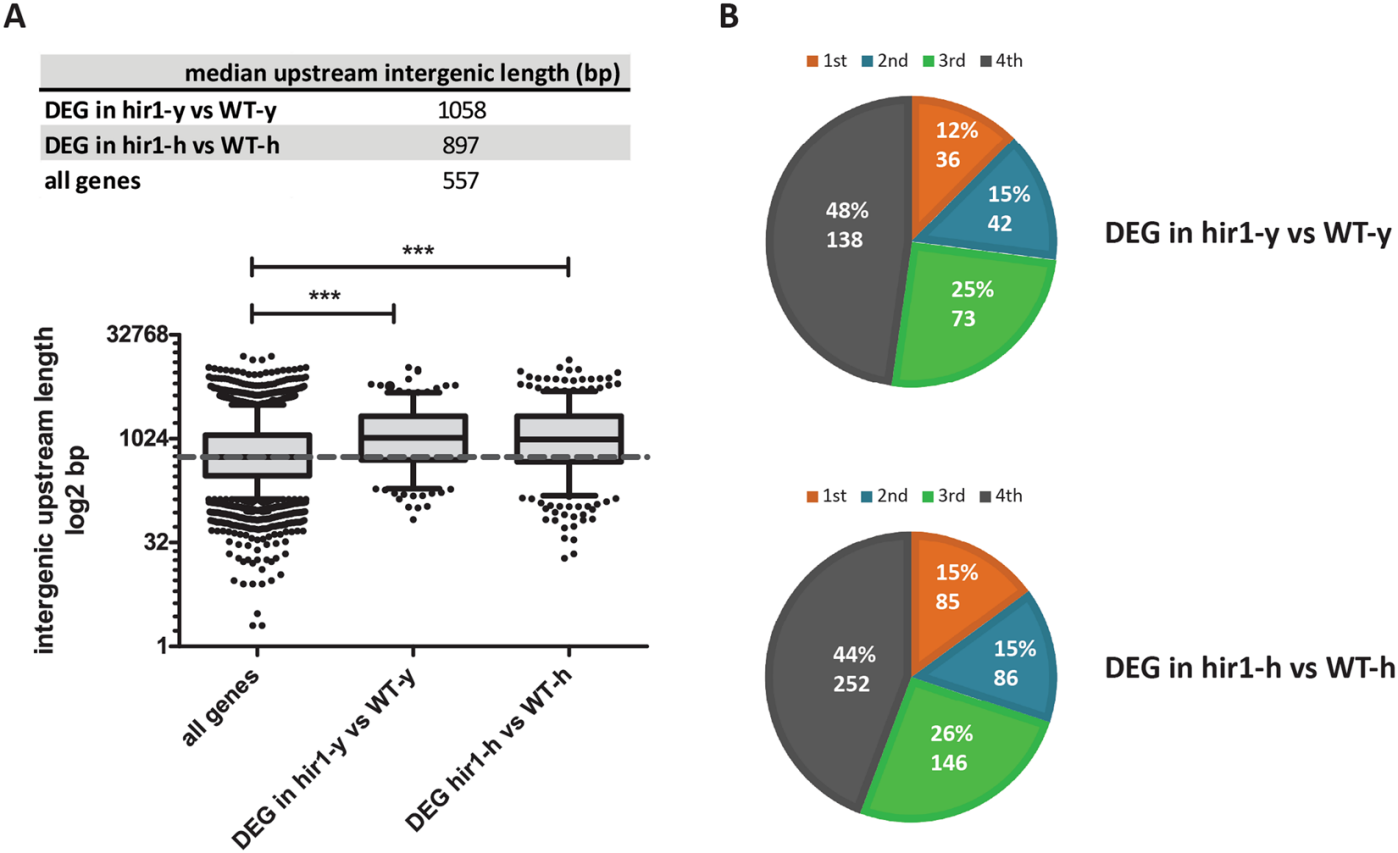
Hir1-affected genes harbor above-average intergenic upstream regions. (**A**) Intergenic upstream regions were extracted from the *C*. *albicans* genome assembly 22 and length distribution of those elements is displayed for all genes detected in the RNA-seq (all genes), for differentially expressed genes (DEGs) in *hir1*∆/∆ cells during yeast growth (hir1-y vs WT-y) and upon hyphal induction (hir1-h vs WT-h). Boxplot whiskers represent the 5–95 percentiles and the dotted line the median upstream intergenic length in “all genes”. Statistical analysis was carried out using Kruskal-Wallis with Dunn’s post hoc test. ***P < 0.001. (**B**) The “all genes” data set was split into quartiles based on the overall upstream intergenic length distribution. The relative gene number and the proportions (%) of DEGs in hir1-y vs WT-y and hir1-h vs WT-h in each quartile are displayed. (**A**,**B**) Differentially expressed genes were defined by an at least 1.5-fold change with an FDR value < 0.05.

In addition to common biological functions of Hir1-regulated genes, we analyzed common genomic sequence elements of gene sets. Of note, previous studies reported unusually long intergenic regions upstream of HSGs in *C*. *albicans* and upstream of developmentally regulated genes in *S*. *cerevisiae* ^57–59^. To evaluate whether Hir1-affected genes harbor unusually long upstream intergenic regions, we analyzed those sequences of genes differentially regulated in *hir1*∆/∆ cells during yeast phase growth (hir1-y) and in response to hyphal stimuli (hir1-h). We found that the median upstream intergenic regions of hir1-y- and hir1-h-affected genes are above-average in size when compared to all expressed genes in our RNA-seq dataset (Fig. 4A). Furthermore, almost 50% of differentially regulated transcripts in hir1-y and hir1-h cells belonged to the top 25% upstream intergenic length quartile (4^th^ quartile) (Fig. 4B). These data provide compelling evidence that Hir1-regulated genes tend to have above-average upstream intergenic regions, which is implying complex transcriptional control by chromatin architecture as well as by a variety of transcriptional regulators^57, 60^.

### *HIR1* deficiency alters the histone densities at the *HWP1* and *UME6* upstream intergenic regions

**Figure 5:**
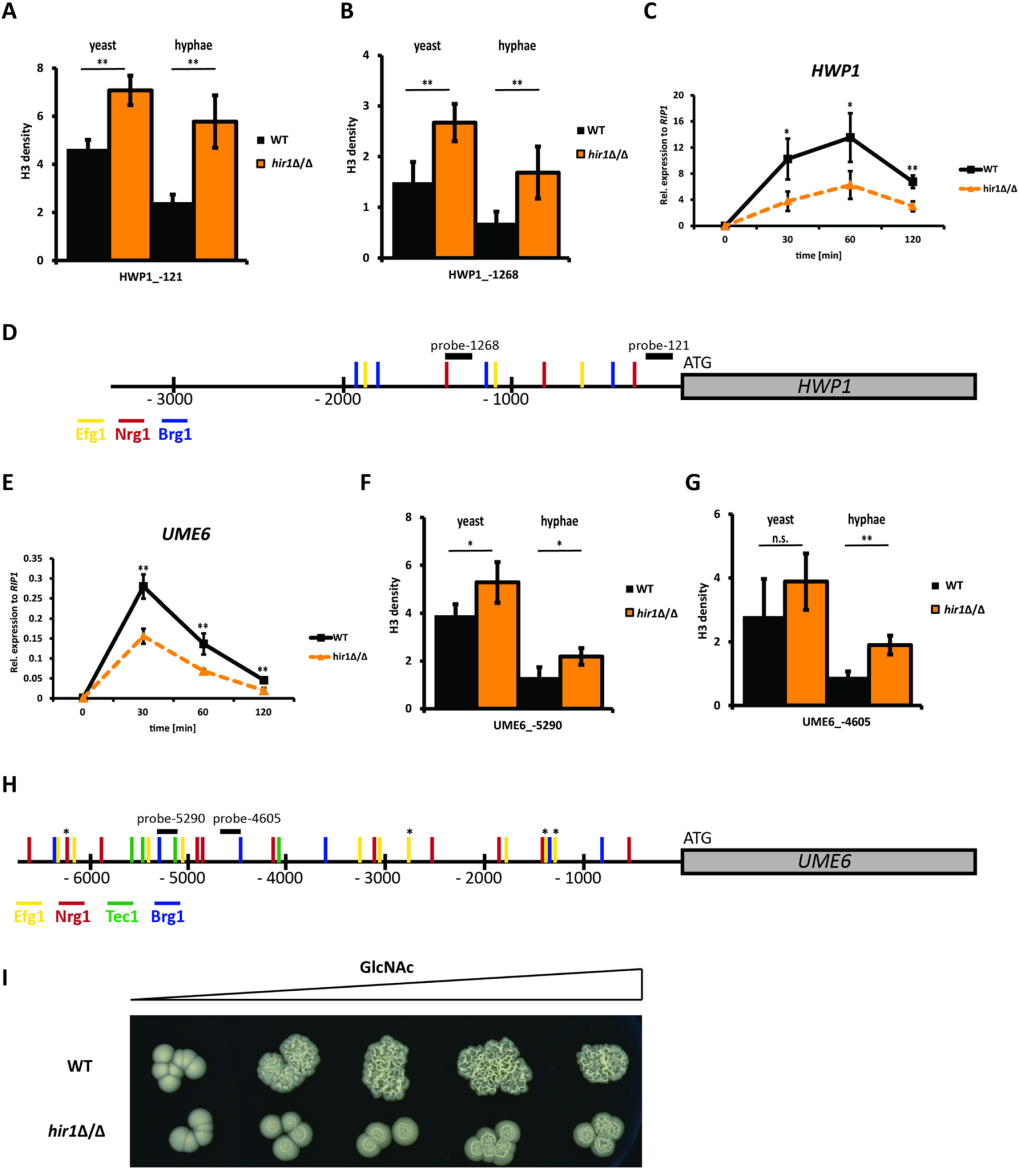
Hir1 affects the chromatin density in the *HWP1* and *UME6* promoter. (**A**,**B**) Loss of *HIR1* increases the histone density in the *HWP1* promoter. Histone density was measured using histone H3 ChIP and qPCR at different *HWP1* promoter regions. (**C**) *HWP1* expression is reduced in *hir1*Δ/Δ cells. WT and *hir1*Δ/Δ cells were grown in YPD with 10% FCS at 37 °C. Cultures were collected at the indicated time points followed by RNA extraction. Gene expression of *HWP1* was measured via RT-qPCR and transcript levels were normalized to the reference gene *RIP1*. (**D**) Representative illustration of transcription factor binding sites in the *HWP1* upstream intergenic sequence. Efg1 (yellow) and Nrg1 (red) binding motifs were taken from ref. 59 and Tec1 (green) and Brg1 (blue) from ref. 13. Note that no putative Tec1 binding site was identified in our *in silico* scan. (E) The same as in (**C**), but for *UME6*. (**F**,**G**) Genetic removal of *HIR1* alters histone occupancy at distinct *UME6* promoter regions. Histone density was measured using histone H3 ChIP and qPCR at different *UME6* promoter regions. (**H**) Putative transcription factor binding sites for the upstream intergenic region of *UME6* are represented as in (**D**). Transcription factor sites with asterisk (*) indicate multiple sites for a given regulator within less than 100 bp. (**I**) *HIR1-*deficient cells require stronger signal intensity to initiate hyphal formation. WT and *hir1*Δ/Δ cells were spotted on YPD supplemented with a continuous GlcNAc concentration gradient of 0–10 mM. Colony morphology was inspected after growth for 3 days at 37 °C. (**A**,**B**,**F**,**G**) The qPCR signals from Input and IP were normalized to an intergenic region on chromosome R. The ratio of normalized Input/IP values is shown on the y-axis labeled as “H3 density”. (**A**–**C**,**E**–**G**): Data are presented as mean + SD of three independent experiments. For significance testing, *hir1*Δ/Δ cells were compared to WT cells. *P < 0.05, **P < 0.01 with Student’s t-test.

Transcriptomic analysis of *hir1*Δ/Δ cells suggested that *HIR1-*deficiency strongly affects the transcriptional amplitudes of regulated genes during hyphal formation. Hir1 and homologues are thought to act in promoter regions of actively transcribed genes to aid deposition of histone H3/H4 dimers^32^. Therefore, we hypothesized that the loss of *HIR1* might result in an altered chromatin state at gene promoters including HSGs. Since histone enrichment in the *HWP1* promoter revealed chromatin dynamics during hyphal formation^16, 17^, and because *HWP1* showed highest induction upon hyphal stimuli in WT and *hir1*Δ/Δ cells (Fig. 3B), we determined histone H3 occupancy in the *HWP1* promoter. The *HWP1* upstream intergenic region of about 2 kb contains several putative binding sites for transcription factors implicated in hyphal formation, including Efg1, Brg1 and Nrg1^17, 59, 61^ (Fig. 5D). Chromatin immunoprecipitation analysis of histone H3 density at the *HWP1* promoter in close proximity to the start codon and near a putative Nrg1 binding site showed a significantly increased histone density in *hir1*Δ/Δ yeast cells when compared to WT yeast cells (Fig. 5A, yeast and Fig. 5D). When cells were subjected to hyphal-inducing conditions, histone levels significantly decreased in the WT, reflecting the onset of *HWP1* transcription. In contrast, histone density was only slightly affected in the mutant (Fig. 5A, hyphae). A similar behavior was observed upstream in the *HWP1* promoter region about 1.25 kb upstream from the translational start site (Fig. 5B, yeast and hyphae). This *HWP1* promoter element has been described as crucial region activating transcription^61^, since it harbors putative binding sites for hyphal activators, including Brg1 and Efg1, as well as for the Nrg1 repressor (Fig. 5D). Based on these results, we hypothesized that altered histone occupancy in the *HWP1* promoter affects the induction rate of transcription of HSGs such as *HWP1*. Indeed, the maximal amplitude of *HWP1* expression was altered in *hir1*Δ/Δ cells, while the overall pattern *per se* was qualitatively similar to WT cells (Fig. 5C).

The *UME6* gene encodes a transcription factor that sustains hyphal induction^60, 62^. Ume6, unlike Hwp1, is essential for hyphal maintenance^63, 64^. Interestingly, the RNA-seq data showed that *UME6* is significantly downregulated in *hir1*Δ/Δ hyphal cells when compared to the WT. This was confirmed by quantifying *UME6* mRNA levels in the WT and *hir1*Δ/Δ cells upon hyphal induction for 2 hours (Fig. 5E). Like *HWP1*, *UME6* expression followed the same kinetics in WT and *hir1*Δ/Δ cells. However, *UME6* transcript induction rate remained lower in *hir1*Δ/Δ cells (Fig. 5E). We then analyzed the histone density of the *UME6* promoter at around 5.3 kb upstream of the start codon. This region harbors a potential binding site for Brg1^60^, Efg1 and Tec1 (Fig. 5H). As seen for the *HWP1* promoter, loss of *HIR1* increased histone occupancy at the *UME6* promoter site in the yeast phase (Fig. 5F, yeast). Upon hyphal induction, WT and *hir1*Δ/Δ cells decreased histone enrichment, whereas *hir1*Δ/Δ hyphal cells retained a significantly higher histone density than the WT (Fig. 5F, hyphae). Inspection of the *UME6* promoter around 4.6 kb upstream of the start codon showed a similar pattern albeit histone density was increased significantly only after hyphal induction in *hir1*Δ/Δ cells (Fig. 5G). This region contains putative binding sites for the Nrg1 repressor, as well as for Brg1 (Fig. 5H). Taken together, these results indicate that the HIR chaperone complex affects the local chromatin density in HSGs already during yeast phase growth, which might impact the recruitment of transcriptional regulators to their target promoters^65^. We identified several putative regulators involved in the control of genes differentially expressed only in *hir1*Δ/Δ yeast or hyphal cells and of genes deregulated in both growth phases (Figure S6A). Thereby, six regulators (Ndt80, Tec1, Sfl2, Fkh2, Mrr1 and Tye7) were associated with all three gene sets. Strikingly, four of these factors, Ndt80, Tec1, Sfl2 and Fkh2, are known regulators of the yeast-to-hyphae transition^13, 66, 67^. Of note, *TEC1* and *SFL2* are downregulated upon the loss of *HIR1* (Figure S5A,B) both in the yeast and hyphal growth phase.

In addition to *HIR1*-affected genes such as *UME6* and *HWP1*, we also assessed histone occupancy in *NRG1* and *CAT1* promoter elements. The transcriptional regulation of these genes did not require Hir1 (as shown in Figures S2 and S3C). We found slightly increased histone levels in the *NRG1* and *CAT1* promoter only upon hyphal induction in *HIR1*-deficient cells (Figure S6B,C), which could be a consequence of general chromatin alterations in the *HIR1* mutant that might not necessarily affect transcription.

Due to increased basal histone densities at the *HWP1*, *UME6* and possibly other gene promoters, the threshold sensitivity to signals required for switching to the hyphal growth phase could be increased in *HIR1*-deficient cells. To test this notion, we spotted WT and *hir1*Δ/Δ cells onto YPD plates containing a GlcNAc concentration gradient as the main filamentation trigger (Fig. 5I). Indeed, the WT was dramatically more sensitive to morphogenetic signals along the entire concentration range of GlcNAc. In contrast, *hir1*Δ/Δ cells required much higher GlcNAc concentrations to initiate hyphal growth. This result provides evidence that, *HIR1* deficiency alters the responsiveness to hyphal growth signals.

### Ectopic expression of *UME6* restores hyphal formation in *hir1*Δ/Δ cells

**Figure 6:**
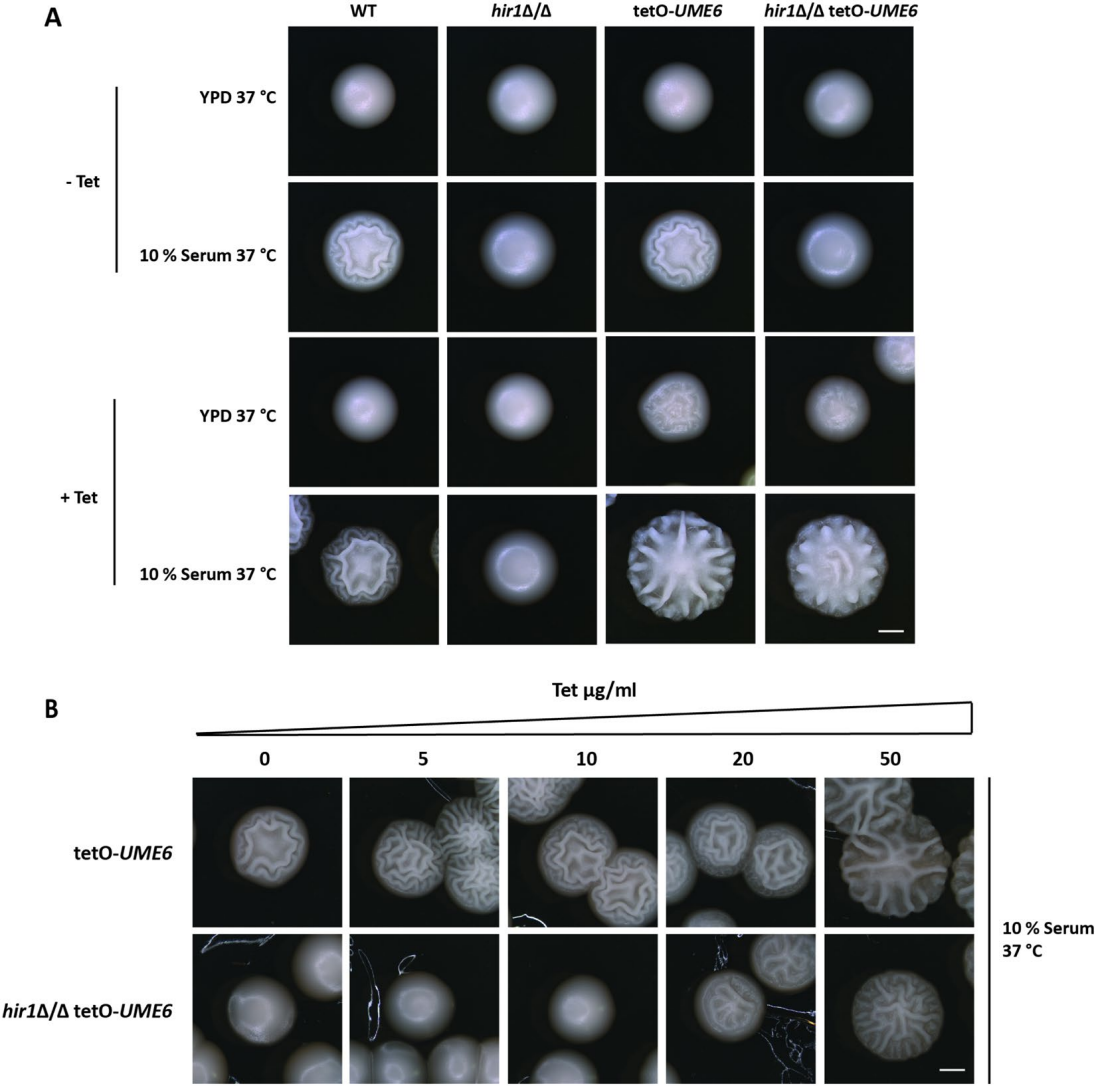
Ectopic expression of *UME6* restores hyphal formation in the absence of *HIR1*. (**A**,**B**) The *UME6* gene was placed under conditional expression control using the tet-ON system^70^. Expression was triggered by adding 50 µg/ml tetracycline (**A**) or various concentrations of tetracycline (**B**). Colony morphology was assessed after growth for 2 days at 37 °C. Scale bar corresponds to 1 mm.

Hyphal induction entails transcriptional activation of circuits acting at different layers. For example, Efg1 is one of the first transcription factors to integrate primary signals, which are transduced to another set of regulators required to maintain HSG expression and hence, filamentation^3, 9, 46^. One of the key downstream regulators is Ume6, which suppresses defective hyphal formation of *efg1*Δ/Δ cells^68, 69^. Our expression and ChIP analysis showed that *UME6* expression was compromised upon genetic removal of *HIR1*. Given that Ume6 is indispensable for sustaining hyphal programs, we investigated whether ectopic overexpression of *UME6* can rescue defective *hir1*Δ/Δ hyphal formation. Therefore, we used the conditional tet-ON system^70^, where *UME6* expression is under the control of a tetracycline-inducible promoter, allowing for *UME6* transcription by adding tetracycline. The tet-ON system had no effect on hyphal formation of WT or *hir1*Δ/Δ cells without tetracycline (Fig. 6A, upper panel). Tetracycline addition increased hyphal formation in WT cells when compared to the respective background control strain on serum-containing YPD at 37 °C (Fig. 6A, lower panel). Strikingly, hyphal induction of the tet-*UME6* expressing *hir1*Δ/Δ cells was fully restored upon tetracycline supplementation. To determine how sensitive *hir1*Δ/Δ cells are to transcriptional induction of *UME6*, we cultivated WT and *hir1*Δ/Δ cells in the presence of different tetracycline concentrations to modulate *UME6* levels. Remarkably, WT cells responded to 5 µg/ml tetracycline and *hir1*Δ/Δ cells required at least a four-fold higher concentration of tetracycline (20 µg/ml) for triggering hyphal induction (Fig. 6B).

In summary, ectopic overexpression of Ume6 rescued filamentation defects of *hir1*∆/∆ cells, suggesting that Hir1 acts at the primary transcriptional valve or molecular clutch, possibly acting in concert with Efg1 upstream of Ume6. These data provide compelling evidence for a tightly interlocked dual-layer regulatory mechanism, which involves both dedicated transcription factors and chromatin architecture to control developmental changes such as morphogenetic cell fate decisions in response to environmental stimuli.

## Discussion

Here, we demonstrate a genetic link between replication-independent chromatin assembly and the transcriptional control of morphogenesis in the human fungal pathogen *C*. *albicans*. We show that Hir1, a subunit of the conserved HIR histone chaperone complex, affects transcriptional amplitudes during the yeast-to-hyphae transition, as activator and repressor of gene expression. The HIR complex is an evolutionary conserved complex initially discovered as a cell cycle-dependent repressor for 6 out of the 8 core histone genes in *S*. *cerevisiae* ^34, 71^. In multicellular organisms, HIR orthologues are implicated in embryonic as well as plant development^72–74^. In *S*. *cerevisiae*, bulk chromatin alterations that affect heterochromatin maintenance, chromosome segregation and DNA damage sensitivity have only been observed in combinatorial deletions of *HIR1* and the CAF-1 complex subunit *CAC2* ^75, 76^. Recently, the conservation of these overlapping functions has been revealed in *C*. *albicans*. It was further demonstrated that Hir1 only affects the morphogenetic opaque to white cell switching upon deletion of the CAF-1 complex subunit *CAC2* ^29^. We previously demonstrated a CAF-1-independent role of Hir1 in antifungal azole tolerance^30^. Here, we explore further novel functions of Hir1 in fungal morphogenesis, which are independent of CAF-1. We reveal a plausible mechanism for HIR-mediated expression control of genes required for cell fate decisions during morphogenetic alterations in *C*. *albicans*.

The ability to undergo and regulate hyphal formation has been viewed as an important virulence factor of *C*. *albicans* ^10, 77^. The main pathway integrating environmental stimuli to drive HSG transcription is the cAMP/PKA pathway, which is, among many other functions, essential for hyphal growth^54, 55, 78^. To translate environmental stimuli into cell-fate changes, this pathway induces a hyphal-specific transcriptional program by activating Efg1 thereby relieving repression by Nrg1^9, 56, 79^. Additionally, earlier reports demonstrated that sustained hyphal growth requires the concerted action of transcriptional activators such as Brg1 and Ume6, the full relief of Nrg1-mediated repression, but also chromatin-associated activities^16, 17^. Thus, at least two regulatory mechanisms execute the yeast-to-hyphae transition, namely hyphal initiation and its maintenance as long as the appropriate signals are present^16^. Our data suggest that Hir1 is involved in the first transcriptional wave, acting downstream of cAMP/PKA signaling. First, although defective in hyphal formation, *hir1*Δ/Δ cells maintain normal cAMP/PKA signaling, since exogenous supplementation of cAMP fails to restore hyphal growth of *hir1*Δ/Δ mutants. Moreover, the PKA subunit Tpk2 and the adenylate cyclase Cyr1 are required for transcriptional downregulation of Nrg1^56^, which is not affected by the loss of *HIR1* following hyphal stimuli. Second, ectopic expression of *UME6*, which is required for hyphal elongation and constitutes a key element of the second transcriptional phase to maintain the morphological stage, can rescue the filamentation defect of *HIR1*-deficient cells, as well as of the *efg1*Δ/Δ mutant^68^. Interestingly, *hir1*Δ/Δ cells hyperfilament under embedded conditions, which closely resembles the lack of *EFG1* and *CYR1* ^80^. Thus, it is tempting to speculate that the action of Hir1 is linked to Efg1-mediated hyphal initiation.

Hir1 is part of a histone chaperone complex, which incorporates histones onto chromatin in a replication-independent manner^32^. Hence, the chromatin architecture at gene promoters could be specifically affected upon the loss of *HIR1*. Here, we show that *hir1*Δ/Δ cells have increased histone levels already during the yeast growth phase at distinct promoter elements of the HSGs *HWP1* and *UME6*. Moreover, both genes displayed elevated histone densities at relevant promoter regions in the *hir1*Δ/Δ mutant in response to hyphal induction, which is reflected in decreased transcript abundance. At this point, we do not know whether increased histone occupancy at these promoters is the cause or the consequence of reduced transcriptional induction of *UME6* or *HWP1*. Moreover, *hir1*Δ/Δ hyphal cells showed slightly increased histone densities at promoter regions of *NRG1* and *CAT1*, for which transcriptional control is unaffected by *HIR1*-deletion. Elevated histone levels in those regions only occurred in response to hyphal induction. Since the HIR complex is a transcriptional repressor of histone genes, we cannot entirely exclude subtle effects of *HIR1*-deficiency on the global chromatin architecture. Given that these effects exist, they most likely do not influence bulk gene transcription, as otherwise expression of the entire genome would become deregulated in *hir1*Δ/Δ cells, which was clearly not the case. Strikingly, in contrast to constitutively expressed genes, highly regulated genes differ in their promoter constellation and often harbor multiple transcription factor binding sites. Hence, they are more likely to be influenced by subtle changes in nucleosome pattern in promoters rather than by the total nucleosome occupancy^81, 82^. Therefore it is plausible that the deregulation of chromatin architecture upon loss of *HIR1* may primarily affect genes with complex regulation. Due to the limited number of loci for which we could inspect nucleosome occupancy, conclusions about a global impact of the HIR complex on chromatin organization are speculative at this point. Thus, further experiments are required to unravel a possible role for the HIR complex in chromatin homeostasis in *C*. *albicans*. However, *in silico* analysis of intergenic upstream regions from Hir1-dependent genes during yeast or hyphal growth showed that those regions are surpassingly long compared to the median length in the *C*. *albicans* genome. Genes implicated in developmental changes such as HSGs often harbor long 5 prime intergenic regions with multiple binding sites for transcriptional regulators, which imply complex transcriptional control, including chromatin remodeling^57, 59^. Hence, it is tempting to speculate that transcription of HSGs, which are regulated by long promoter regions, are more likely to be subject to transcriptional de-regulation when chromatin homeostasis is disturbed. This potentially impacts early transcriptional fine-tuning following hyphal induction stimuli. Defective transcriptional amplitudes can then be compensated by enhancing upstream signaling intensity for hyphal induction. Thus, the interplay of chromatin state and transcriptional control could provide a platform to efficiently adjust the sensitivity towards stimulating cues, and thus, modulate transcriptional threshold levels required for developmental changes or environmental responses.

Here we show that although *hir1*Δ/Δ cells induce the hyphal-specific transcriptional program in response to hyphal triggers, efficient hyphal formation is impaired due to altered amplitudes of this response. Indeed, we demonstrate that *HIR1*-deficient cells are less sensitive to hyphal-inducing conditions and that this defect can be compensated by stronger trigger for hyphal development or by ectopic overexpression of the *UME6* transcription factor for hyphal maintenance. Therefore, it is tempting to speculate that Hir1 affects initial fine-tuning of transcription by assisting in homeostasis of the chromatin template and thereby safe-guarding the transition from the yeast to the hyphal growth phase. A similar mechanism has been shown for the mammalian homologue of Hir1, HIRA, which deposits the histone variant H3.3 preferentially at gene promoters^83^. In a mouse oogenesis model, HIRA is required for normal chromatin homeostasis and transcriptional fine-tuning. Similarly, binding of the polycomb repressive complex 2 (PRC2) to developmental genes upon differentiation stimuli depends on HIRA-mediated H3.3 deposition in mouse embryonic stem cells^84^.

Hir1/HIRA may be recruited via different components, including transcription factors^84, 85^, RNA Polymerase II and potentially even naked DNA^86^. We identified six transcription factors (Ndt80, Tec1, Sfl2, Fkh2, Mrr1 and Tye7) with target genes differentially regulated only in *hir1*Δ/Δ yeast or hyphal cells and also genes that are differentially expressed in both growth phases. Interestingly, Ndt80, Tec1, Sfl2 and Fkh2 are implicated in hyphal formation and *TEC1* and *SFL2* expression seems to depend on Hir1 action, since both genes are downregulated upon *HIR1*-deletion. Further experiments are still required in order to reveal to what extent HIR modulates the affinity of transcription factors or the general transcription machinery to HSGs. Since only half of all differentially expressed genes in the *hir1*Δ/Δ mutant are altered in a hyphal-specific context, it will be of great interest to investigate how Hir1 affects the chromatin architecture and to unravel the precise mechanism of Hir1 recruitment to target genes in *C*. *albicans*. Of note, we were unable to perform ChIP experiments with the Hir1-myc tagged strain to show a direct interaction of Hir1 with HSG promoters. However, this could be explained by transient nature of interactions, insufficient cross-linking of Hir1 and target DNA caused by a possible as yet unknown intermediate regulatory factor recruiting Hir1, as well as by high off rates of regulators.

Taken together, our study provides evidence for the necessity of the tight control of gene expression amplitudes upon encounter of changing environmental conditions exemplified here with the *C*. *albicans* yeast-to-hyphae transition. The HIR complex seems to contribute to the regulation of the initial phase of hyphal formation, where it might control signal sensitivity by acting as a valve or clutch that controls transcriptional loads via chromatin homeostasis. This way, the HIR complex could assist in the swift execution of responses to stimuli, and in the case of *C*. *albicans*, ascertains rapid adaptions to changing host environments. Importantly, the replication-independent nature of HIR function offers a major advantage for fungal cells, since it enables adaptive changes in gene expression without involving excessive and energy-intensive replication or cell cycle control. Hence, further exploring the general relevance of HIR-dependent transcriptional regulation will provide exciting new insights into *C*. *albicans* pathophysiology and host adaption as well as fungal virulence.

## Materials and Methods

### Media, chemicals and growth conditions, colony morphology and microscopy

Rich medium (YPD) was prepared as described previously^87^. Spider medium contained 1% Bacto yeast extract (BD Biosciences, Vienna, Austria), 1% mannitol (Merck, Vienna, Austria), 0.2% KH2PO4, 2% histidine, 2% leucine (all Sigma-Aldrich, Vienna, Austria) and 2% Bacto agar (BD Biosciences) for solid medium. *C*. *albicans* strains were routinely grown on YPD at 30 °C. For hyphal induction, a fresh saturated overnight culture was harvested, washed once with distilled water, resuspended in an equal volume corresponding to the starting overnight culture and diluted 1:10 in pre-warmed fresh YPD containing 10% fetal calf serum (FCS; serum)^39^. These cultures were then further grown at 37 °C for the indicated periods of time. Cultures were either used directly for further analysis or snap-frozen at −80 °C.

To investigate hyphal induction on solid medium, overnight cultures were diluted to an OD_600_ of 0.1 in fresh YPD and then propagated to the logarithmic growth phase. Cells were counted on a CASY® cell counter and approximately 50 (10 cm dishes) or 10 (6 cm dishes) cells were plated on YPD agar plates supplemented with FCS, N-acetyl glucosamine (GlcNAc), 3′,5′-Cyclic adenosine monophosphate (cAMP) (all Sigma-Aldrich) or Spider medium.

Colony morphology and microscopic inspection of single cells was performed as described previously^22^. For quantification of the yeast and hyphal fraction of a population, at least 200 cells were counted.

### Plasmid and strain constructions

The *C*. *albicans* strains, primers and plasmids used in this study are listed in Table S1. All *C*. *albicans* strains were derived from the *MTL* **a**/α clinical isolate SC5314^88^. The strains lacking *HIR1*, *CDC35*, *CAC2*, *RTT106*, *CPH1* and *EFG1* have been reported earlier^22, 30, 89^.

The *HPC2* and *HIR3*-deficient mutants were generated using a modified *SAT1*-flipper technique^90^. Briefly, approximately 500 bp up- and downstream sequences of the target gene ORF were PCR amplified and cloned into the plasmid pSFS3b using ApaI, KpnI and BglII, NotI, respectively. Deletion of *NRG1* was done in a similar way using the plasmid pSFS2a^91^. The plasmid for *HIR2* deletion was constructed by fusing approximately 500 bp up- and downstream region of the target gene, the FLP-FRT-*NAT1*-FRT marker from pSFS3b and the pSFS3b backbone by an *in vivo* cloning approach in *E*. *coli* EL350 as described in ref. 92. Ectopic overexpression of *UME6* was accomplished by the tet-ON system using the pNIM1 plasmid described earlier^70^. Gene-complemented strains were generated by cloning the *HIR1* ORF with ApaI and KpnI upstream of the FLP recombinase and *NAT1* into pSFS3b. 9myc epitope-tagging of *HIR1* and *NRG1* alleles was carried out by fusion PCR^93^ using plasmid pFA6a-9myc-*NAT1* as a template. Correct genomic integration of the deletion cassette and re-integration constructs was verified with colony PCR, Southern Blot (for *HIR1* knock-out, 9myc-tagged and complementation strain only) and/or immunoblotting (for epitope-tagged strains only).

### Immunoblotting, immunoprecipitation and mass spectrometry analysis

Whole-cell-free extracts and immunoblotting was performed exactly as described earlier^90^. For detection of Nrg1-9myc, a mouse monoclonal anti-myc antibody (clone 4A6, MFPL monoclonal antibody facility, Vienna, Austria) was used. An anti-PSTAIRE antibody recognizing Cdc28 (Santa Cruz Biotechnology, Santa Cruz, CA) was used as a protein loading control. Protein bands on the nitrocellulose membrane were visualized using an Odysee® CLx scanner (Li-Cor®, Lincoln, NE). Cells were prepared for immunoprecipitation exactly as described previously^90^. Whole-cell extracts corresponding to 50 OD_600_ units were incubated with 4 µl of the monoclonal anti-myc antibody. After an overnight incubation at 4 °C, 30 µl of Protein-G-coupled Dynabeads (Invitrogen, Vienna, Austria) were added for 2 hours at 4 °C. Subsequent washing steps were performed as described for the ChIP experiments, except that PBS instead of 1xTE was used for the final washing steps. The beads were then resuspended in Laemmli buffer for SDS-PAGE analysis. Silver staining of 10% SDS-PAGE gels was performed essentially as described elsewhere^94^. To identify resolved proteins, protein bands were cut out from the silver-stained gel, digested with trypsin and separated using an LC system for subsequent mass spectrometry analysis. Mass spectrometry analysis was carried out at the mass spectrometry facility at the Vienna Biocenter. The raw spectra were matched against the *Candida albicans* genome database (http://www.candidagenome.org/).

### Chromatin immunoprecipitation (ChIP)

ChIP was performed essentially as described earlier^37^. For histone ChIP, 1 mg cell free protein extract was incubated with 1 µl of anti-Histone H3 antibody (#1791, Abcam, Cambridge, UK). After overnight incubation at 4 °C, 30 µl of Protein-G-coupled Dynabeads (Invitrogen) were added for 2 hours at 4 °C. Subsequent washing steps and DNA purification were performed as described previously using minor modifications^95^. Input and ChIP DNA were incubated with 5 µl of 10 mg/ml DNase-free RNase A at 65 °C for 16 hours and recovered using a MiniElute^®^ PCR Purification Kit (Qiagen, Hilden, Germany). Input DNA was quantified on a NanoDrop-2000 spectrophotometer (Peqlab, Erlangen, Germany) and ChIP DNA concentration was measured using the Quantifluor^®^ dsDNA System (Promega, Mannheim, Germany) in accordance to the manufacture’s protocol. Histone density at the *HWP1* promoter was analyzed using primers amplifying the region ranging from −243 to −106 and from −1299 to −1195 with respect to the start codon. To analyze the *UME6* promoter region, primers ranging from −4534 to −4676 and from −5210 to −5371 relative to the start codon were used. For the *NRG1* and *CAT1* promoter, a region ranging from −1702 to −1502^37^ and −162 to −306^30^, respectively, was inspected. Input and IP qPCR signals were normalized to an intergenic region on chromosome R (RT5_tC_inter and RT3_tC_inter primers), before calculating the Input/IP ratio. The displayed “H3 density” in Figs 5A,B,F,G and S6B,C represents the enrichment over Input relative to the aforementioned intergenic region.

### RNA isolation, RT-qPCR analysis, RNA-seq analysis and bioinformatics

RNA isolation, cDNA synthesis and qPCR analysis was performed exactly as described previously^90^. Relative quantification of mRNA levels was done by qPCR using the efficiency corrected ΔΔCt method^96^. *RIP1* was used as a reference gene^22^. Statistical analysis was performed in Excel (Microsoft). DNase-treated RNA was subjected to rRNA depletion using the RiboMinus^TM^ Eukaryote System v2 (Life Technologies, Vienna, Austria) adhering to the recommended protocol. The efficiency of rRNA depletion was checked using a Bioanalyzer with a RNA6000 Nano chip (Agilent, Vienna, Austria). Fragmentation of rRNA-depleted RNA was done using the NEBNext Magnesium RNA fragmentation module (New England BioLabs, Vienna, Austria) following the manufactures instructions. Samples were incubated at 94 °C for 4 minutes in a thermocycler. Fragmented samples were recovered using the RNeasy MiniElute Cleanup Kit (Qiagen) and subjected to 1^st^ strand synthesis using SuperScript III Reverse Transcriptase (Life Technologies) and 3 µg random primers (Life Technologies, Vienna, Austria). Mini Quick Spin columns (Qiagen) were used to clean up the single-stranded cDNA, which was subsequently subjected to 2^nd^ strand synthesis using the NEBNext mRNA Second Strand Synthesis Module (New England BioLabs) following the manual. Additionally, T4 DNA polymerase (New England BioLabs) was added for the last 5 minutes to polish overhanging ends. Double-stranded cDNA was cleaned up using a MiniElute PCR Purification Kit (Qiagen). DNA concentration was measured using the PicoGreen® dsDNA quantification reagent (ThermoFisher Scientific, Vienna, Austria).

Double-stranded cDNA was further processed and sequenced on a HiSeq. 2500 instrument (Illumina, San Diego, CA) by the Vienna Biocenter Campus Support Facility CSF (NGS unit, http://www.csf.ac.at). Two biological replicates for the WT and the *hir1*Δ/Δ cells during yeast growth or hyphal induction were subjected to RNA-seq analysis.

RNA-seq reads were mapped to the *C*. *albicans* genome Assembly 21 using TopHat, allowing for only uniquely mapped reads^97^. All reads mapping to rRNA loci were removed. Read counts were determined with HTSeq using the union mode^98^ and a reference annotation (C_albicans_SC5314_version_A21-s02-m07-r10; http://www.candidagenome.org). Differential gene expression was analyzed using edgeR^99^. P-values were adjusted to determine differentially regulated genes^100^. Venn diagrams were created by Venny 2.0.2 (http://bioinfogp.cnb.csic.es/tools/venny/index.html)^101^. GO analysis was carried out with FungiFun2 (https://elbe.hki-jena.de/fungifun/fungifun.php)^102^. The whole dataset of detected protein-coding genes and their expression can be found in Table S2.

### *In silico* upstream intergenic sequence analysis

Sequence information for all *C*. *albicans* ORFs plus intergenic up- and downstream sequences were retrieved from the *Candida* genome database (CGD; http://www.candidagenome.org; C_albicans_SC5314_version_current_orf_plus_intergenic.fasta accessed on April 24^th^, 2017). Putative transcriptional regulators of differentially expressed genes in *HIR1*-deficient yeast and hyphal cells were identified using the PathoYeastract Database “Rank by TF” tool, allowing only for hits with verified target binding and expression data^103^.

### Data availability

The authors confirm that all data underlying the findings are fully available without restrictions. All relevant data are within the paper and its Supporting Information files.

## Electronic supplementary material

Supplementary Information

Table S2
