# Supplementary material for: The Candida albicans HIR histone chaperone regulates the yeast-to-hyphae transition by controlling the sensitivity to morphogenesis signals
Source: Sci Rep. 2017 Aug 16;7:8308. doi: 10.1038/s41598-017-08239-9 (PMC5559454; doi:10.1038/s41598-017-08239-9)
Supplement: Supplementary file 1 — Supplementary Information [file 41598_2017_8239_MOESM1_ESM.doc]

**Supporting information: The *Candida albicans* HIR histone chaperone regulates the yeast-to-hyphae transition by controlling the sensitivity to morphogenesis signals**

Sabrina Jenull, Michael Tscherner, Megha Gulati, Clarissa J. Nobile, Neeraj Chauhan & Karl Kuchler


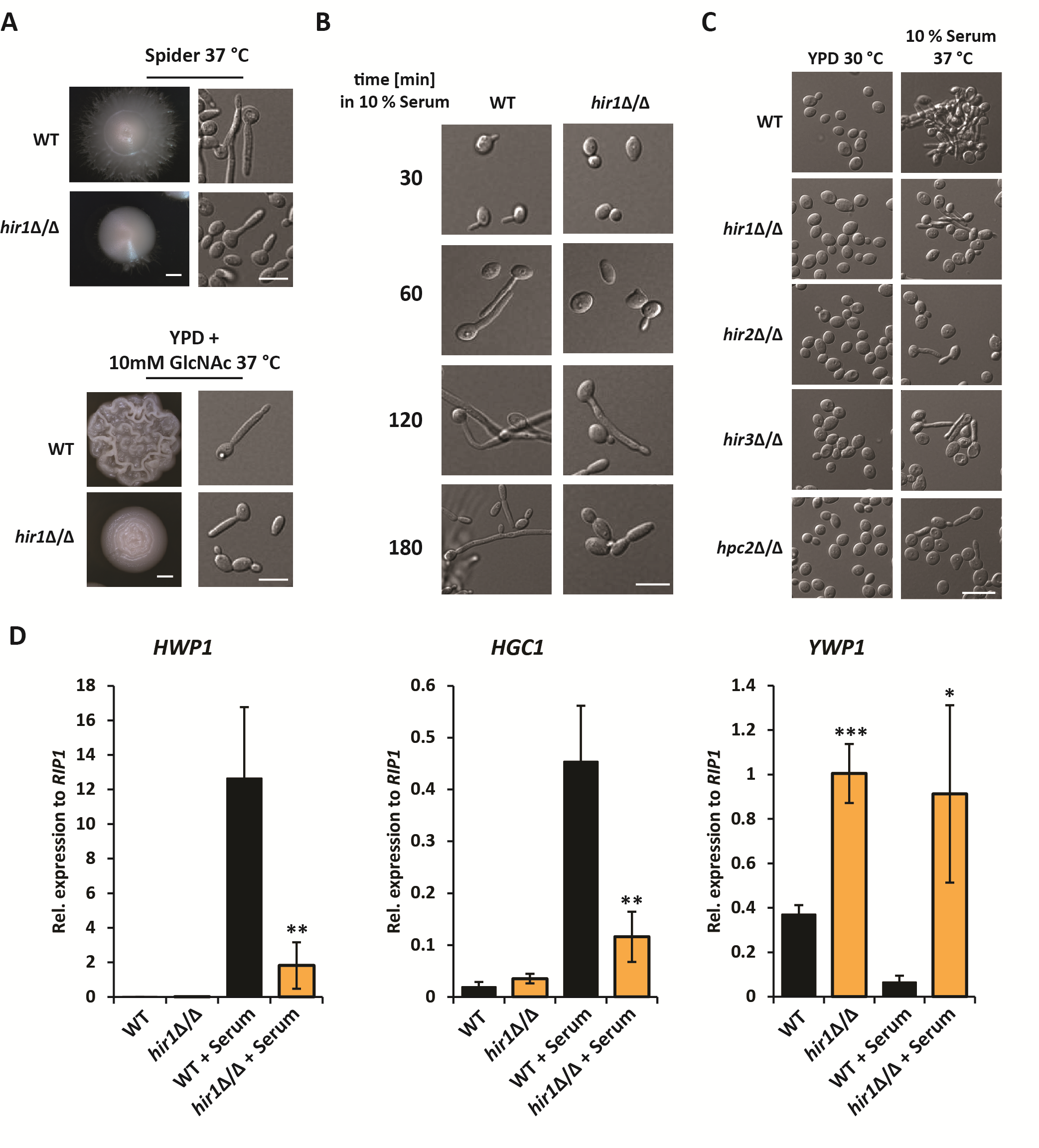


**Figure S1: Hir1 controls hyphal formation in response to various stimuli.** (A) *hir1*Δ/Δ mutants show decreased hyphal formation in different hyphal-inducing liquid and solid media. In liquid medium, WT or *hir1*Δ/Δ mutant cells were grown in hyphal-inducing conditions. Differential Interference Contrast (DIC) pictures from fixed cultures were taken after 2 hours of incubation at 60x magnification in an Olympus IX81 microscope. Colony morphology was assessed on solid media after 3 days at 37°C on YPD containing 10mM GlcNAc or on Spider medium. Scale bar corresponds to 1 mm on solid medium and 20 µm in liquid medium. (B) Representative pictures from time course experiment shown in Fig. 1B. (C) HIR complex mutants have decreased hyphal formation in liquid YPD with 10% FCS at 37°C. Pictures were taken as in (A). Scale bar corresponds to 20 µm. (D) Other hyphal-activated or –repressed genes in WT or *hir1*Δ/Δ cells from the experiment shown in Fig. 1C. For significance testing, *hir1*Δ/Δ cells were compared to WT cells. *P<0.05, **P<0.01, ***P<0.001 with Student’s t-test.


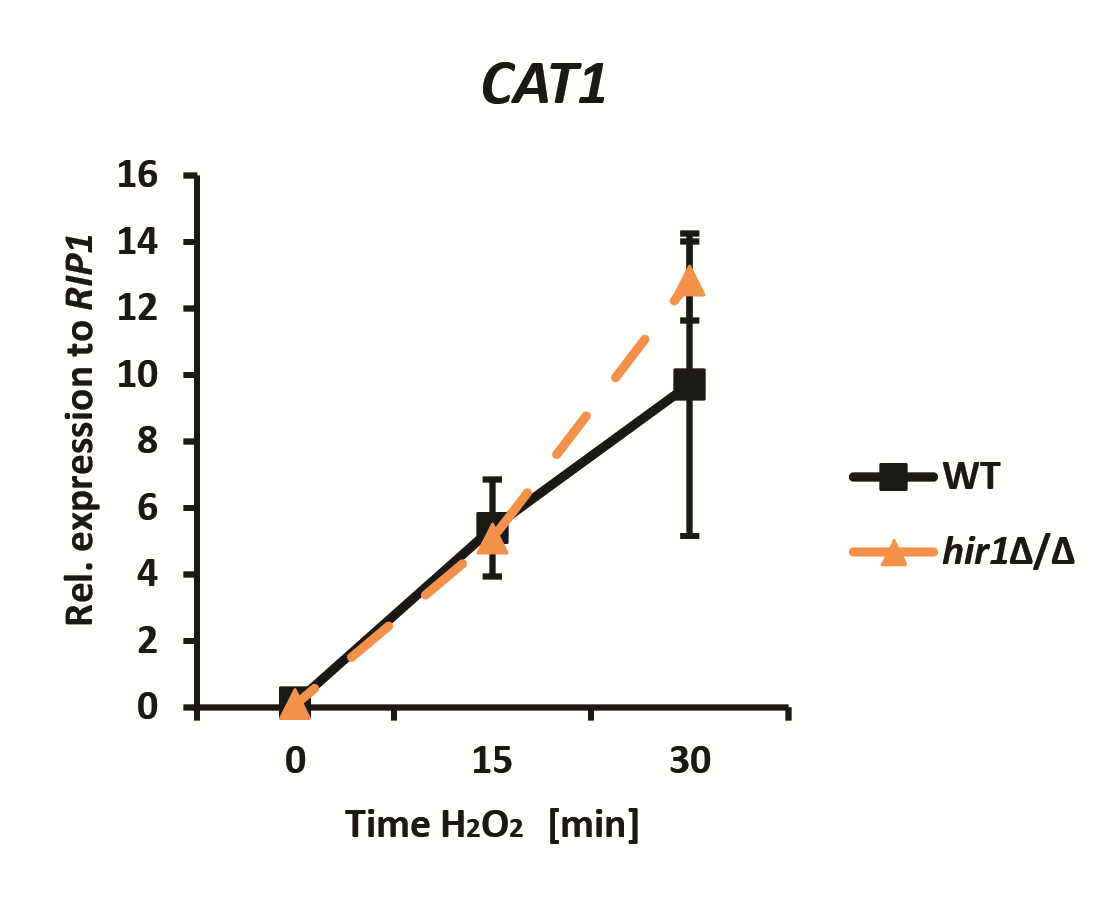


**Figure S2: WT and *HIR1—*deficient cells respond equally to oxidative stress.** The indicated strains were grown to log-phase in YPD at 30°C. Oxidative stress was induced by the addition of 1.6mM H_2_O_2_. Cultures were collected at the indicated time following RNA extraction. *CAT1* gene expression was quantified using RT-qPCR and levels were normalized to *RIP1*. Data are presented as the mean + SD from three independent experiments.


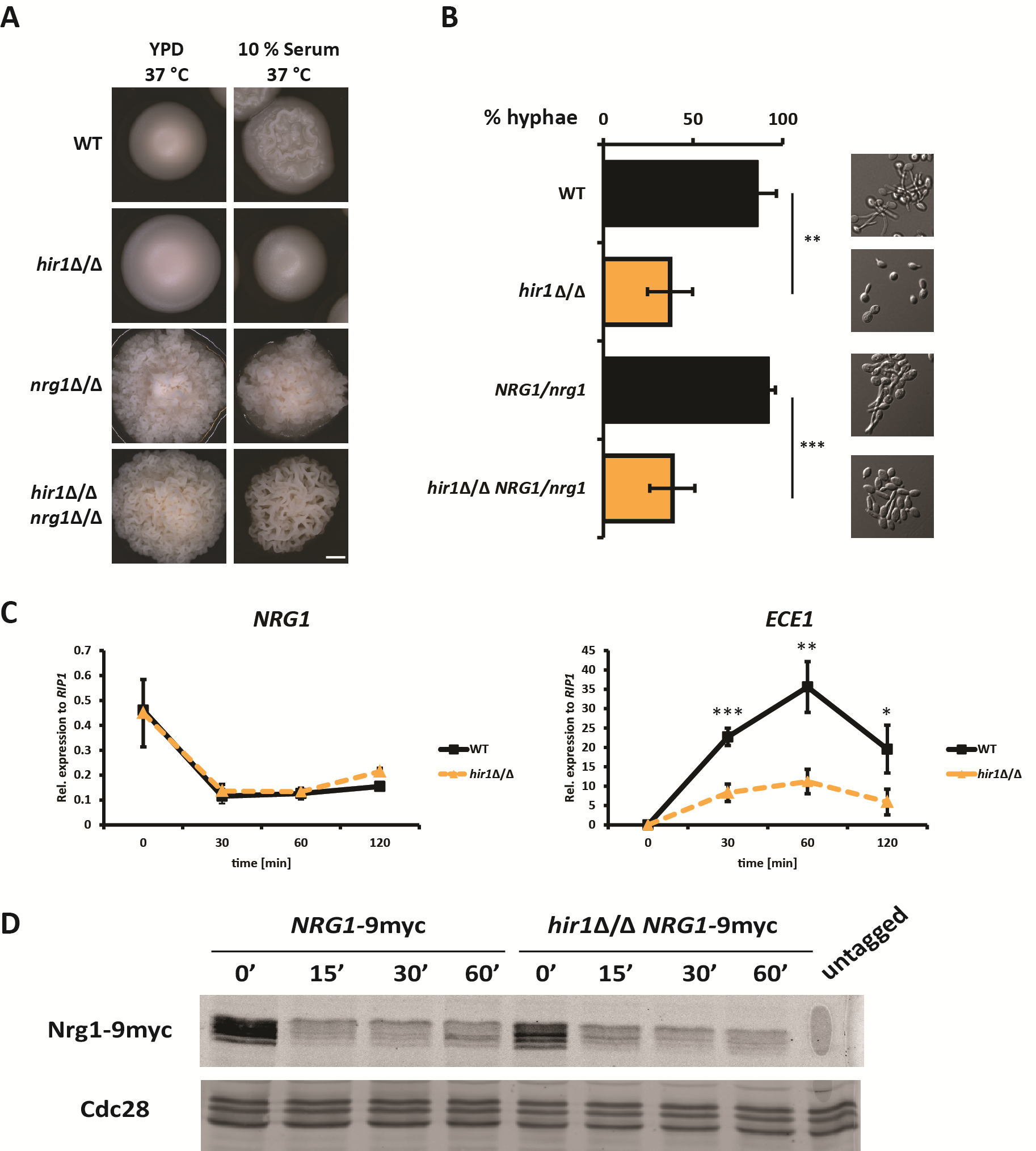


**Figure S3: The effect of *NRG1* dosage on *hir1*Δ/Δ hyphal formation.** (A) Loss of *NRG1* rescues the *hir1*Δ/Δ filamentation defect. Colony morphology was inspected after 3 days at 37°C. Scale bar corresponds to 1 mm. (B) Reduction of *NRG1* gene dosage does not alter *hir1*Δ/Δ hyphal formation in liquid medium. The indicated strains were induced to form hyphae in YPD with 10% FCS at 37°C for 60 min. Cultures were fixed and hyphal formation was imaged and quantified. Representative pictures are shown on the right side of the graph. (C) *NRG1* downregulation does not require *HIR1*. WT and *hir1*Δ/Δ cells were grown in YPD with 10% FCS at 37°C. Cultures were collected at the indicated time following RNA extraction. Gene expression of *NRG1* and *ECE1* was measured via RT-qPCR and levels were normalized to the reference gene *RIP1*. (D) Loss of *HIR1* does not affect steady-state Nrg1 protein levels. Nrg1 was epitope-tagged with 9myc in either WT or *hir1*Δ/Δ cells and subjected to hyphal induction in YPD with 10% FCS 37°C. Cultures were collected at the indicated time and subjected to protein extraction for immunoblotting. Nrg1-9myc was detected using an anti-myc monoclonal antibody (clone 4A6). The untagged *NRG1/nrg1* strain served as a control. Cdc28 was used as protein loading control. (B-C): Data are presented as the mean + SD of three independent experiments. For significance testing, *hir1*Δ/Δ cells were compared to WT cells. *P<0.05, **P<0.01, ***<P0.001 with Student’s t-test.


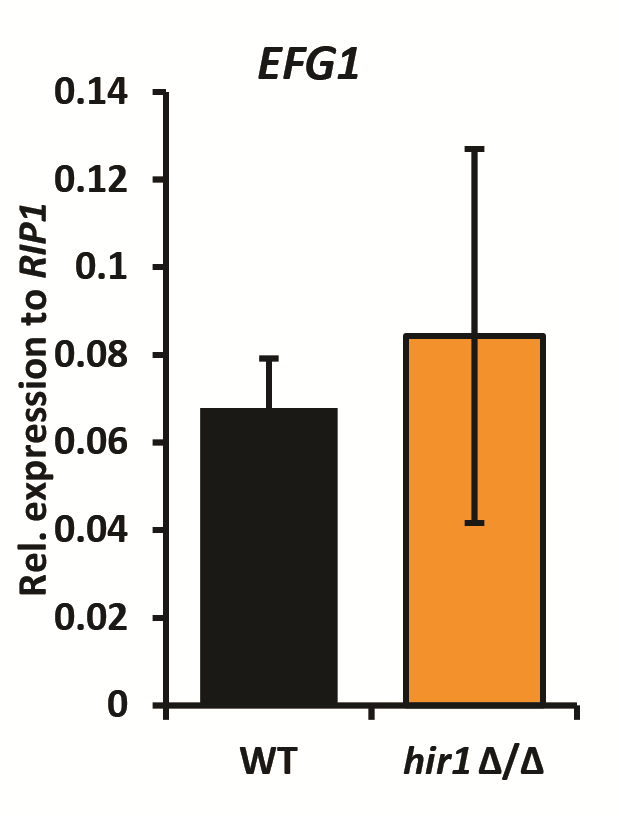


**Figure S4: Basal transcription of *EFG1* is not affected by *HIR1* deletion.** Transcript levels of *EFG1* were quantified as in Fig. 1C during yeast morphology growth. Data are presented as mean + SD of four independent experiments.


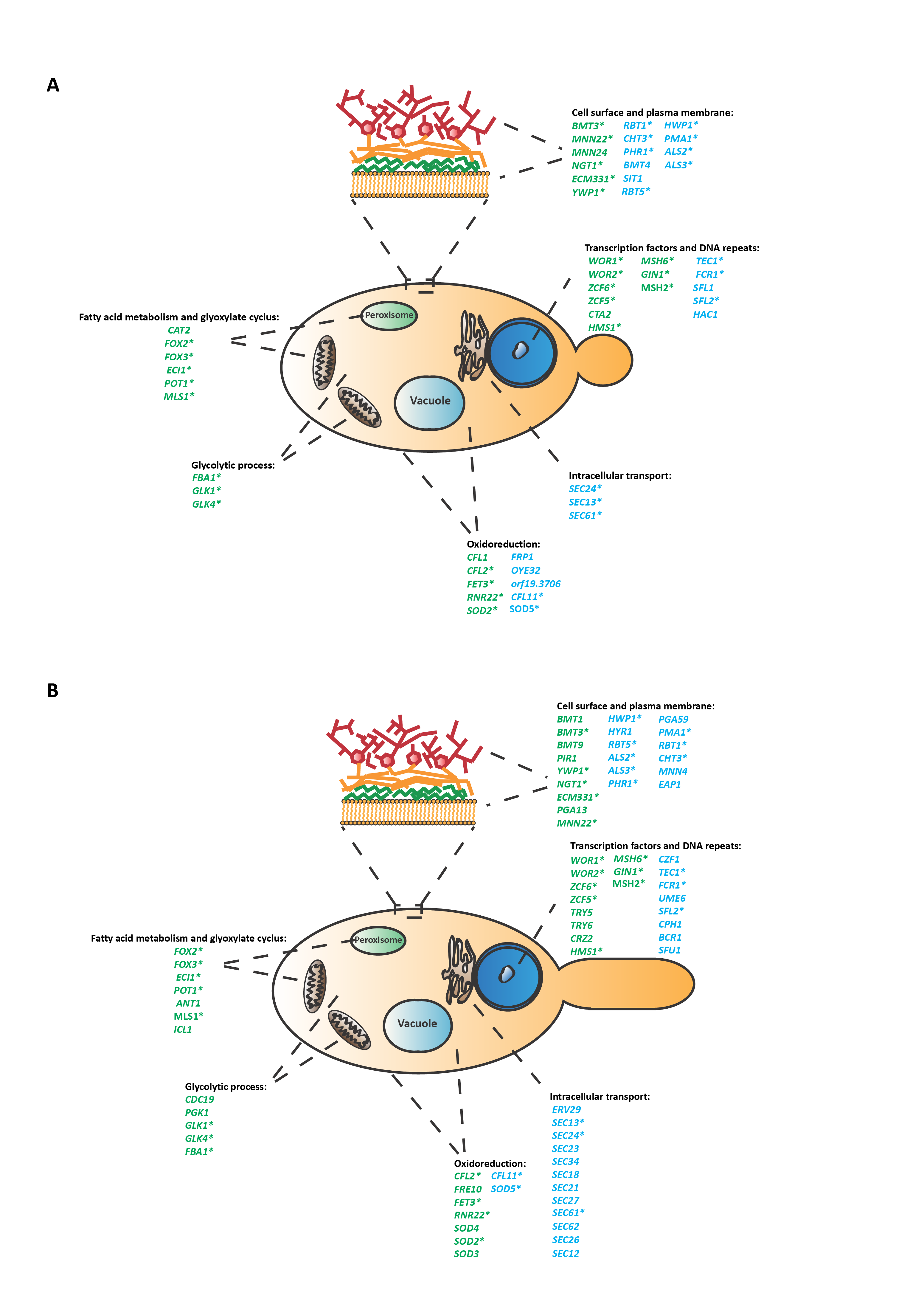


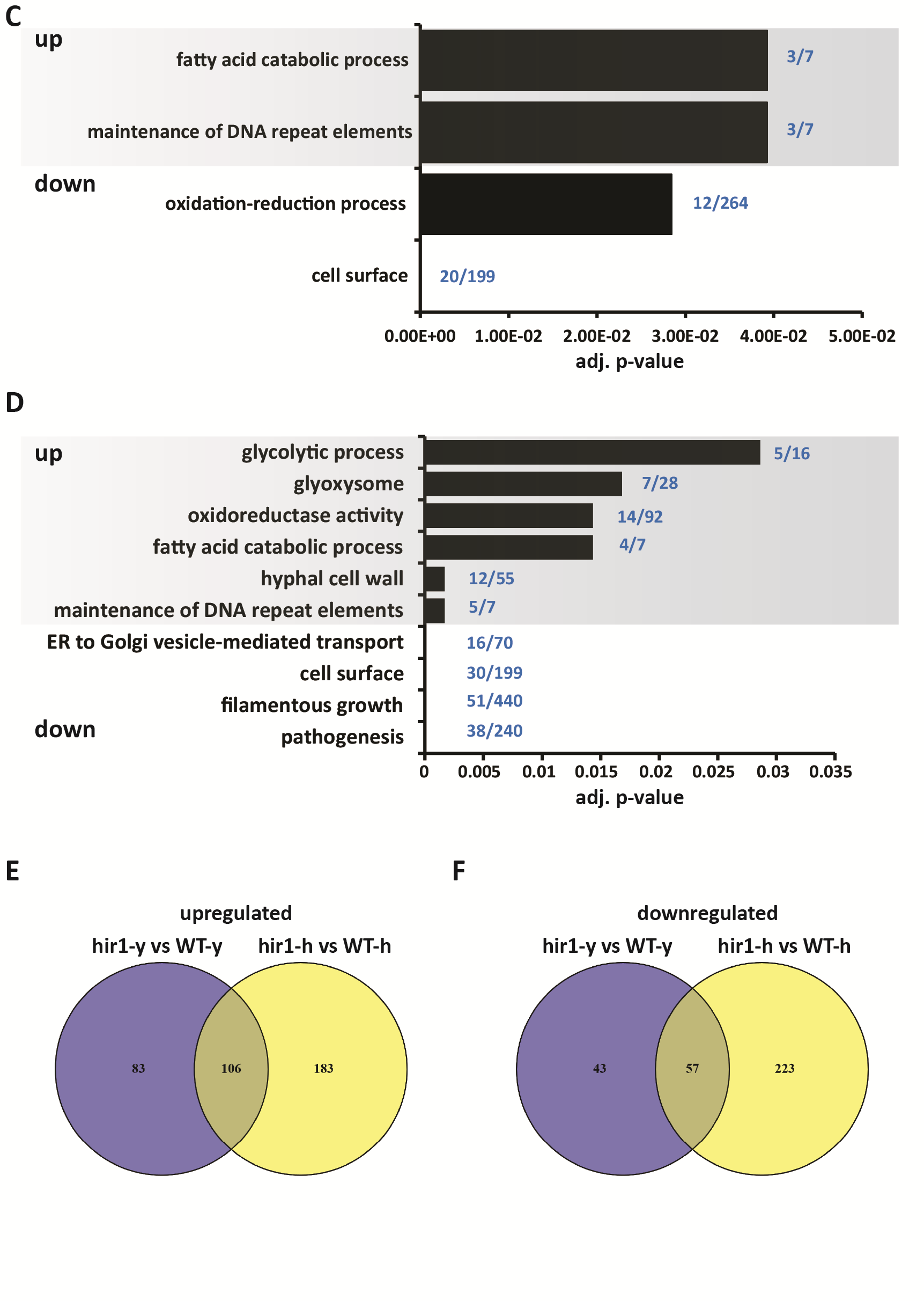


**Figure S5: Hir1 affects various biological processes.** Loss of *HIR1* impacts various cell compartments and biological processes during yeast growth (A) as well as hyphal initiation (B). Gene names presented in green were upregulated and gene names in blue were downregulated in *hir1*Δ/Δ cells when compared to WT cells. The corresponding biological processes are derived from GO term analysis or gene description in the *Candida* genome database (CDG) (http://www.candidagenome.org). Gene names marked with an asterisk (*) were differentially expressed in *hir1*Δ/Δ yeast and hyphal cells. (C-D) GO-term enrichment analysis of differentially expressed genes in *hir1*Δ/Δ yeast cells vs WT yeast cells (C) or in *hir1*Δ/Δ hyphal cells vs WT hyphal cells (D). Blue numbers indicate the number of genes from the dataset/total number of genes in the GO-term group. (E-F) Overlap of up- (E) or downregulated (F) genes in the *hir1*Δ/Δ mutant growing in the yeast (y) and hyphal phase (h) when compared to the WT. (A-F): differentially expressed genes were defined by an at least 1.5-fold change and FDR < 0.05.


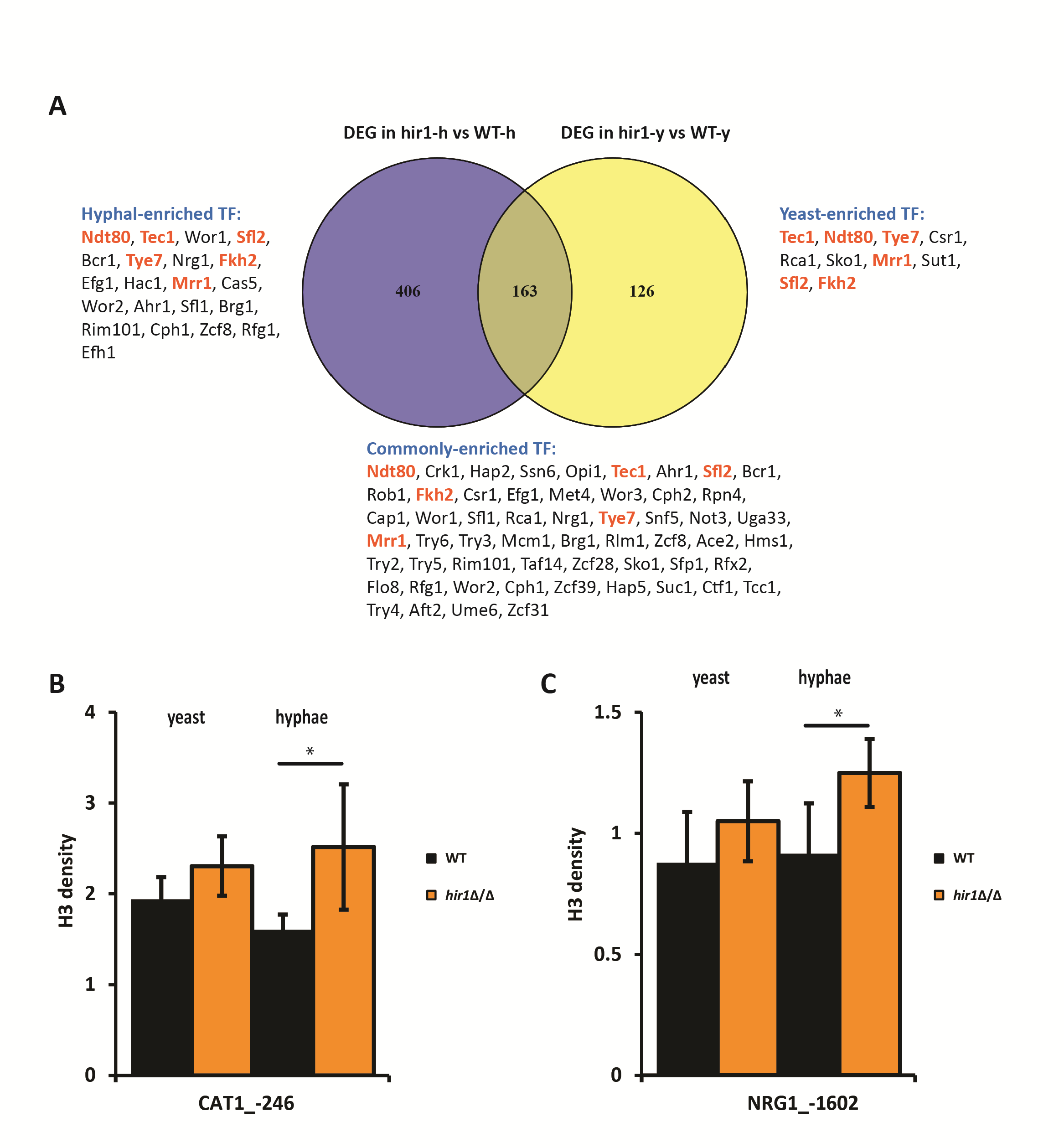


**Figure S6: Hir1-affected genes.** (A) Putative transcriptional regulators of Hir1-affected genes. Putative regulatory transcription factors (TFs) of differentially expressed genes (DEGs) in *HIR1-*deficient cells during yeast growth (hir1-y vs WT-y; yellow circle), upon hyphal induction (hir1-h vs WT-h; violet circle) or under both conditions (grey intersection of the circles) have been identified using the *C. albicans* PathoYeastract [^1^](#_ENREF_1) “Rank by TF” tool. TFs displayed in orange have target genes in all three gene sets (“commonly-enriched TF”; A). (B-C) Loss of *HIR1* exerts subtle effects on the histone density at Hir1-independent genes upon hyphal induction. Histone density at *CAT1* and *NRG1* promoter regions (approximately -246bp and -1602bp upstream from the ATG start codon of the *CAT1* and *NRG1* CDS, respectively) was determined as in Figure 5 using ChIP-qPCR. Data are presented as mean + SD of three independent experiments. For significance testing, *hir1*Δ/Δ cells were compared to WT cells. *P<0.05 with Student’s t-test.

**Table S1. *C. albicans*** strains, plasmids and primers used in the study.

| **Description** | **Name** | **Parent** | **Genotype** | **Reference** | **Figure** |
| --- | --- | --- | --- | --- | --- |
| WT | SC5314 | Clinical isolate |  | [^2^](#_ENREF_2) | Figure 1-2; 5-6; S1-3; S5 |
| *hir1*Δ/Δ | CA-MT376 | SC5314 | *hir1*::FRT/*hir1*::FRT | [^3^](#_ENREF_3) | Figure 1-2; 5-6; S1-3; S5 |
| *hir1*Δ/Δ::*HIR1* | CA-SJ013 | SC5314 | *hir1*::FRT/*hir1*::*HIR1*-FRT | This study | Figure 1 |
| *hir2*Δ/Δ | CA-SJ105 | SC5314 | *hir2*::FRT/*hir2*::FRT | This study | Figure 1; S1 |
| *hir3*Δ/Δ | CA-SJ009 | SC5314 | *hir3*::FRT/*hir3*::FRT | This study | Figure 1; S1 |
| *hpc2*Δ/Δ | CA-SJ060 | SC5314 | *hpc2*::FRT/*hpc2*::FRT | This study | Figure 1; S1 |
| Hir1-9myc | CA-MT366 | SC5314 | *hir1*::FRT/*hir1::HIR1*-9myc-*NAT1* | This study | Figure 1 |
| Hir1-9myc  *hpc2*Δ/Δ | CA-SJ128 | SC5314 | *hir1*::FRT/*hir1::HIR1*-9myc-*NAT1* *hpc2*::FRT/*hpc2*::FRT | This study | Figure 1 |
| Hir1-9myc  *hir2*Δ/Δ | CA-SJ153 | SC5314 | *hir1*::FRT/*hir1::HIR1*-9myc-*NAT1 hir2*::FRT/*hir2*::FRT | This study | Figure 1 |
| Hir1-9myc  *hir3*Δ/Δ | CA-SJ144 | SC5314 | *hir1*::FRT/*hir1::HIR1*-9myc-*NAT1* *hir3*::FRT/*hir3*::FRT | This study | Figure 1 |
| *NRG1*/*nrg1* Δ | CA-MT184 | SC5314 | *NRG1*/*nrg1*::FRT | This study | Figure 2; S3 |
| *cac2*Δ/Δ | CA-MT363 | SC5314 | *cac2*::FRT/*cac2*::FRT | [^3^](#_ENREF_3) | Figure 1 |
| *rtt106*Δ/Δ | CA-MT408 | SC5314 | *rtt106*::FRT/*rtt106*:FRT | [^3^](#_ENREF_3) | Figure 1 |
| *nrg1*Δ/Δ | CA-MT200 | SC5314 | *nrg1*::FRT/*nrg1*::FRT | This study | Figure S3 |
| *hir1*Δ/Δ *NRG1*/*nrg1*Δ | CA-SJ051 | SC5314 | *hir1*::FRT/*hir1*::FRT *NRG1*/*nrg1*::FRT | This study | Figure 2 |
| *hir1*Δ/Δ *nrg1*Δ/Δ | CA-SJ065 | SC5314 | *hir1*::FRT/*hir1*::FRT *nrg1*::FRT/*nrg1*::FRT | This study | Figure S3 |
| Nrg1-9myc | CA-SJ055 | SC5314 | *nrg1*::FRT/*nrg1*::*NRG1*-9myc-*NAT1* | This study | Figure S3 |
| *hir1*Δ/Δ  Nrg1-9myc | CA-SJ056 | SC5314 | *hir1*::FRT/*hir1*::FRT  *nrg1*::FRT/*nrg1*::*NRG1*-9myc-*NAT1* | This study | Figure S3 |
| *efg1*Δ/Δ | DHCA216 | SC5314 | *efg1*::FRT/*efg1*::FRT | [^4^](#_ENREF_4) | Figure 2 |
| *cph1*Δ/Δ | JKC19 | SC5314 | *ura3::λimm^434^*/*ura3::λimm^434^* *cph1*::*hisG*/*cph1*::*hisGURA3hisG* | [^5^](#_ENREF_5) | Figure 2 |
| *efg1*Δ/Δ  *NRG1*/*nrg1* | CA-SJ157 | SC5314 | *efg1*::FRT/*efg1*::FRT *NRG1*/*nrg1*::*NAT1* | This study | Figure 2 |
| *cdc3*5Δ/Δ | DHCA442 | SN152 | *cdc35*::*C.d*.*HIS1*/*cdc35*::*C.m.LEU2* | [^4^](#_ENREF_4) | Figure 2 |
| tetO-*UME6* | CA-SJ149 | SC5314 | *ADH1*/*adh1*::P_tet_-*UME6* | This study | Figure 6 |
| *hir1*Δ/Δ tetO-*UME6* | CA-SJ151 | SC5314 | *hir1*::FRT/*hir1*::FRT *ADH1*/*adh1*::P_tet_-*UME6* | This study | Figure 6 |

**A. Strains used in this study**

B. Plasmids used in this study

| **Plasmid** | **Parent** | **Insert** | **Reference** |
| --- | --- | --- | --- |
| pSFS3b |  |  | [^6^](#_ENREF_6) |
| pSFS2a |  |  | [^7^](#_ENREF_7) |
| pFA6a-9myc-*NAT1* |  |  | [^6^](#_ENREF_6) |
| pNIM1 |  |  | [^8^](#_ENREF_8) |
| pSFS3b-*HIR1*rev | pSFS3b | *HIR1*-FRT-FLP-*NAT1*-FRT | This study |
| pSFS3b-*HIR3*urdr | pSFS3b | 5’*HIR3*-FRT-FLP-*NAT1*-FRT-3’*HIR3* | This study |
| pSFS3b-*HIR2*urdr | pSFS3b | 5’*HIR2*-FRT-FLP-*NAT1*-FRT-3’*HIR2* | This study |
| pSFS3b-*HPC2*urdr | pSFS3b | 5’*HPC2*-FRT-FLP-*NAT1*-FRT-3’*HPC2* | This study |
| pSFS2a-*NRG1*urdr | pSFS2a | 5’*NRG1*-FRT-FLP-*SAT1*-FRT-3’*NRG1* | This study |
| pNIM1-*UME6* | pNIM1 | *UME6* | This study |

**C. Oligonucleotide p**rimers used in this study

| **Name** | **Sequence (5’->3’)** |
| --- | --- |
| **Gene deletion and 9myc-tagging constructs based on the *SAT1*-flipping technique** | |
| SATflipp_fwd | ATGACCATGATTACGCCAAGC |
| SATflipp_rev | CGACTCACTATAGGGCGAATTGG |
| CAT_fwd | GAAGCATAAAGTGTAAAGCCTG |
| CAT_rev | atcgtcaattattacctccac |
| 55Tag_HIR1 | TTATGTGTTGTGCTCGTTCCATG |
| 53Tag_HIR1 | ccactagcagcagaaccggaTATCAAATCAAGTAGTTCTTGACCTC |
| 9myc_HIR1_oe_fwd | GAGGTCAAGAACTACTTGATTTGATAtccggttctgctgctagtgg |
| pFA6a-backb_rev | ccgacaccggtcgacctgcagcaGCAGGTTAACCTGGCTTATCG |
| 35Tag_HIR1 | gctgctgcaggtcgaccggtgtcggCCTTTGTATCTCTAACTGGCTTTG |
| 33_HIR1_NotIApaI | actgggcccgcggccgcCCATTGGGAAATCCACAATCCAAG |
| 55_Ca7150 | gggcccTCTTCTTTTTGAGCGAGTTCG |
| 53_Ca7150 | ctcgaGATTGTTTGATTCTTAATGAAACTAGC |
| 35_Ca7150 | gcggccgcGTGGTCAAAATTGTATTATAAAACGG |
| 33_Ca7150 | ctcgagctcAAATAATATAGAACACTTATGCCACG |
| 55_tag NRG1 | GTACCGATTATAATCAGACCATC |
| 53_tag NRG1 | ccactagcagcagaaccggaTACTAGGCTCTTGGTGTTGT |
| 35_tag NRG1 | tgctgcaggtcgaccggtgtcgGGATGGTTAATTGCTTGGG |
| 33_NRG1 | TTTCATCGTGTTACAATGCTAGG |
| 5_9myc_NRG1 ov | ATCACCCCAAGTTCAGTCATtccggttctgctgctagtgg |
| 55_HIR2 | gctcaggctctccccgtggaggtaataattgacgatGTGATGGAAATGGCTTCTTAGTTG |
| 53_HIR2 | ctcgagggggggcccggtacccaattcgccctatagtgagtcgGCCAATAGTAGTAAGAGTTCAGATC |
| 35_HIR2 | tagtgagggttaattgcgcgcttggcgtaatcatggtcatGTTAAATGGAGACCAACACCG |
| 33_HIR2 | ctcactcattaggcaccccaggctttacactttatgcttcGATATTAATCCACCGGGTATACCA |
| 55_HPC2_ApaI fo | agtcgggcccCACTTGAACAGCTTACCTTATGTG |
| 53_HPC2_KpnI re | agtcggtaccCAGAAGCCATCAGTTAAATTCG |
| 35_HPC2_BglII fo | agtcagatctGGTAGGTGATAGTACATACATACG |
| 33_HPC2_NotIKpnI re | agtcgcggccgcggtaccCCTTCTTTGATTGGAGTCTATAAGC |
| 55_HIR3_ApaI fo | agtcgggcccGTATAGAAGAGGAATTGAGTGG |
| 53_HIR3_KpnI re | agtcggtaccCCATTCCTTATGGTTAGCTCA |
| 35_HIR3_BglII fo | agtcagatctAGCCATCAATTGACTTCACC |
| 33_HIR3 NotIKpnI re | agtcgcggccgcggtaccGAACGAGTCCACAACAAATTC |
| **Primers to verify genomic integration** | |
| 5C_HPC2 | TCTGTTGTTCAGACATGCAC |
| 3C_HPC2 | GGTATGAATTCATTAAGGACTCAC |
| 5C_HIR2 | ACATATTCCTCGTTAGCCATC |
| 3C_HIR2 | CTTGGATAAATTGGATCGAATC |
| 5C_HIR3 | TCGGTTTGGAAGTTGGGTTTATC |
| 3C_HIR3 | GACTAGAATGCAAACAGAGTCA |
| 5C_tag NRG1 | TCATATCAACAACGTCACCAAC |
| 3C_tag NRG1 | CTGGAAATGTAACGTCACCTG |
| 5C_NRG1 | GCTTGGAAAAATGCCTTCATGG |
| 3C_NRG1 | CTGCCATTGGTGTTTCATCG |
| **Primers to verify loss-of-gene for gene deletion mutants** | |
| HPC2_LOG_fo | ATCCTAAAGATCCACAACCTGG |
| HPC2_LOG_re | TGGAATTTCTATCGTCTACCAATG |
| HIR2_LOG_fo | AGTGGTGGTAGCAACAATAAAC |
| HIR2_LOG_re | TGAACCAATACTCTTTGAGCATC |
| HIR3_LOG_fo | ATTTACCGAGAACTTGATCAACAC |
| HIR3_LOG_re | CTCTTGTGACAAATTGTCGTCATC |
| **qPCR Primers** | |
| RT5_ECE1 | TGCCATTTGTTGTCAGAGCTG |
| RT3_ECE1 | TAGCTTGTTGAACAGTTTCCAGG |
| RT5_HWP1 | GCTGGTTCAGAATCATCCATGC |
| RT3_HWP1 | AAGGTTCAGTGGCAGGAGCTG |
| RT5_HGC1 | GTCAGCTTCCTGCACCTCATC |
| RT3_HGC1 | AAACAGCACGAGAACCAGCG |
| RT5_NRG1 | GGTTGCACGTTGTCGAAACC |
| RT3_NRG1 | TGTTGCTGCTGCTGCTTGG |
| RT5_RHD1 | GGATGAAGAAGATTGTGTTTGGG |
| RT3_RHD1 | ATTGACTAATTCAGTACCACCACG |
| RT5_YWP1 | TGCTAGTACTGCTAACAAAGTCAC |
| RT3_YWP1 | CACCATTAACACCACCAGCA |
| RT5_UME6 | TCATTCAATCCTACTCGTCCACC |
| RT3_UME6 | CCAGATCCAGTAGCAGTGCTG |
| RT5_CAT1_2 | GAGACCCATCTAAATTCCCAC |
| RT3_CAT1_2 | CTTCATTGCTAGTCAAGTAATCCC |
| RT5_RIP1 | TGCTGACAGAGTCAAGAAACC |
| RT3_RIP1 | GAACCAACCACCGAAATCAC |
| RT5_HWP1_-121 | CGTTTTTGCAACTTCTCTTTGTATC |
| RT3_HWP1_-121 | GAAAATAGCCGGTTATTTTGACCGT |
| RT5_HWP1_-1268 | CAAGAAATACAGGAAACCCTCC |
| RT3_HWP1_-1268 | CATATCGTATGTTTCAAAGAG |
| RT5_UME6_-5290 | CATTCCACAACAAACGGTAAGAG |
| RT3_UME6_-5290 | ACACCTAGCGTGAGTAGTAGAAG |
| RT5_UME6_-4605 | TGTGGTTAATGCAGCTTGTCTTG |
| RT3_UME6_-4605 | AGCAGGAGGTAAAGCAGGAAG |
| CAT1_-306_-280 | TTTCCCATTTCCTTTAATAGTTATAG |
| CAT1_-162_-186 | AATTTTATTGGTAACTTTCAAGGG |
| CJNO1410 (*NRG1*pro) | AGTCAATTATGTTGCACAAATCCCG |
| CJNO1411 (*NRG1*pro) | ACTCTTCTTGAATCTAAACAAACCTATAAAACC |
| RT5_EFG1 | CATCACAACCAGGTTCTACAACCAAT |
| RT3_EFG1 | CTACTATTAGCAGCACCACCC |
| RT5_tC_inter | GTGCTATTTACATTCGGTCTTGTTG |
| RT3_tC_inter | TGGGGATGCACAAGATGTAAGTG |

**Table S2. RNA-seq dataset (as .xls)**

**Supplementary references**

1 Monteiro, P. T. *et al.* The PathoYeastract database: an information system for the analysis of gene and genomic transcription regulation in pathogenic yeasts. *Nucleic acids research* **45**, D597-D603, doi:10.1093/nar/gkw817 (2017).

2 Gillum, A. M., Tsay, E. Y. & Kirsch, D. R. Isolation of the Candida albicans gene for orotidine-5'-phosphate decarboxylase by complementation of S. cerevisiae ura3 and E. coli pyrF mutations. *Molecular & general genetics : MGG* **198**, 179-182 (1984).

3 Tscherner, M. *et al.* The Candida albicans Histone Acetyltransferase Hat1 Regulates Stress Resistance and Virulence via Distinct Chromatin Assembly Pathways. *PLoS pathogens* **11**, e1005218, doi:10.1371/journal.ppat.1005218 (2015).

4 Hnisz, D. *et al.* A histone deacetylase adjusts transcription kinetics at coding sequences during Candida albicans morphogenesis. *PLoS genetics* **8**, e1003118, doi:10.1371/journal.pgen.1003118 (2012).

5 Liu, H., Kohler, J. & Fink, G. R. Suppression of hyphal formation in Candida albicans by mutation of a STE12 homolog. *Science* **266**, 1723-1726 (1994).

6 Tscherner, M., Stappler, E., Hnisz, D. & Kuchler, K. The histone acetyltransferase Hat1 facilitates DNA damage repair and morphogenesis in Candida albicans. *Molecular microbiology* **86**, 1197-1214, doi:10.1111/mmi.12051 (2012).

7 Reuss, O., Vik, A., Kolter, R. & Morschhauser, J. The SAT1 flipper, an optimized tool for gene disruption in Candida albicans. *Gene* **341**, 119-127, doi:10.1016/j.gene.2004.06.021 (2004).

8 Park, Y. N. & Morschhauser, J. Tetracycline-inducible gene expression and gene deletion in Candida albicans. *Eukaryotic cell* **4**, 1328-1342, doi:10.1128/EC.4.8.1328-1342.2005 (2005).
